# A cell-free antigen processing system informs HIV-1 epitope selection and vaccine design

**DOI:** 10.1084/jem.20221654

**Published:** 2023-04-14

**Authors:** Srona Sengupta, Josephine Zhang, Madison C. Reed, Jeanna Yu, Aeryon Kim, Tatiana N. Boronina, Nathan L. Board, James O. Wrabl, Kevin Shenderov, Robin A. Welsh, Weiming Yang, Andrew E. Timmons, Rebecca Hoh, Robert N. Cole, Steven G. Deeks, Janet D. Siliciano, Robert F. Siliciano, Scheherazade Sadegh-Nasseri

**Affiliations:** 1 Department of Medicine, Johns Hopkins University School of Medicine, Baltimore, MD, USA; 2 Department of Pathology, Johns Hopkins University School of Medicine, Baltimore, MD, USA; 3 Department of Inflammation and Oncology and Genome Analysis Unit, Amgen Research, Amgen Inc., South San Francisco, CA, USA; 4 Department of Biological Chemistry, Mass Spectrometry and Proteomics Facility, Johns Hopkins University School of Medicine, Baltimore, MD, USA; 5 Department of Biology, Johns Hopkins University, Baltimore, MD, USA; 6 Department of Medicine, University of California, San Francisco, San Francisco, CA, USA; 7 Howard Hughes Medical Institute, Baltimore, MD, USA; 8 The Graduate Program in Immunology and Medical Scientist Training Program, Johns Hopkins University School of Medicine, Baltimore, MD, USA

## Abstract

Distinct CD4^+^ T cell epitopes have been associated with spontaneous control of HIV-1 replication, but analysis of antigen-dependent factors that influence epitope selection is lacking. To examine these factors, we used a cell-free antigen processing system that incorporates soluble HLA-DR (DR1), HLA-DM (DM), cathepsins, and full-length protein antigens for epitope identification by LC-MS/MS. HIV-1 Gag, Pol, Env, Vif, Tat, Rev, and Nef were examined using this system. We identified 35 novel epitopes, including glycopeptides. Epitopes from smaller HIV-1 proteins mapped to regions of low protein stability and higher solvent accessibility. HIV-1 antigens associated with limited CD4^+^ T cell responses were processed efficiently, while some protective epitopes were inefficiently processed. 55% of epitopes obtained from cell-free processing induced memory CD4^+^ T cell responses in HIV-1^+^ donors, including eight of 19 novel epitopes tested. Thus, an in vitro processing system utilizing the components of Class II processing reveals factors influencing epitope selection of HIV-1 and represents an approach to understanding epitope selection from non–HIV-1 antigens.

## Teaser

This study uses a cell-free antigen processing system that mimics major histocompatibility complex Class II processing to reveal factors influencing epitope selection across the HIV-1 proteome. Novel epitopes are identified, and epitopes revealed from cell-free processing are primed in vivo.

## Graphical Abstract

**Figure:**
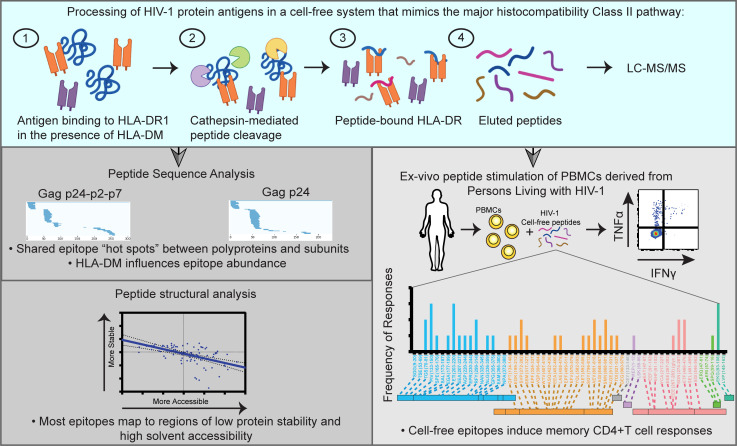

## Introduction

CD4^+^ T cells connect the humoral- and cell-mediated arms of the immune system, both of which are vital for vaccine responses against chronic viral infections. Elegant analyses of CD4^+^ T cell responses to HIV-1 by Walker and colleagues have provided insights into protective HLA-DR alleles and viral epitopes associated with control of viral replication. The breadth and magnitude of Gag-specific CD4^+^ T cell responses are associated with anti-Env neutralizing antibodies (Ranasinghe et al., 2015) and inversely correlated with viral load (Laher et al., 2017; Ranasinghe et al., 2012; Ranasinghe et al., 2013). Three CD4^+^ T cell epitopes in Gag are associated with spontaneous viral control (Ranasinghe et al., 2012, 2013). However, it remains unclear why few individuals develop these protective responses and whether this is influenced by antigen-processing mechanisms.

The antigen-processing pathway for major histocompatibility complex Class II (MHC-II)–restricted CD4^+^ T cell epitopes begins with the endocytosis of exogenous antigens or autophagy of intracellular contents (Unanue et al., 2016). Full-length protein antigens bind to MHC-II molecules in the late endosomal MHC-II compartment (MIIC) of professional APCs, with subsequent cleavage/trimming of the exposed protein around the bound region (Kim et al., 2014). Epitope selection by MHC-II is facilitated by chaperones HLA-DM (DM) and HLA-DO. DM recognizes structurally flexible conformations of peptide:MHC-II (pMHC-II) complexes (Chou and Sadegh-Nasseri, 2000) due to partially filled or unfilled P1 pockets in the peptide binding groove (Anders et al., 2011; Chou and Sadegh-Nasseri, 2000) and induces dissociation of poorly bound peptides such as the class II–associated invariant chain (CLIP). The resulting open MHC-II groove is peptide-receptive and can rapidly scan for the best fitting sequences (Chou and Sadegh-Nasseri, 2000; Natarajan et al., 1999; Rabinowitz et al., 1998), generating tightly formed pMHC-II complexes that are no longer recognized by DM (Narayan et al., 2007, 2009) and that would likely have a longer half-life on the surface of APCs (Nelson et al., 1994). DO works cooperatively with DM to promote its function in B cells, where it is primarily expressed (Poluektov et al., 2013; Welsh et al., 2019, 2020).

CD4^+^ T cell epitopes are often defined using overlapping synthetic peptides. While this approach provides a broad survey of the T cell epitopes within proteins of interest, it does not reveal the parameters that determine epitope selection. This is because the antigen-processing steps needed to generate pathogen-derived pMHC-II complexes are bypassed. CD4^+^ T cell responses are the final output in a long series of steps following infection and are influenced by antigen-dependent and independent factors. Antigen-dependent factors include structural features of the native protein, the molecular context of the epitope within the protein (Kim and Sadegh-Nasseri, 2015; Kim et al., 2017; Mirano-Bascos et al., 2008), the affinity of the epitope for the relevant MHC molecule (Yewdell and Bennink, 1999), resistance to DM-mediated editing of the pMHC-II complex (Kim and Sadegh-Nasseri, 2015) leading to the selection of stable pMHC-II (Lazarski et al., 2005), and TCR affinity for pMHC-II (Malherbe et al., 2004). Antigen-independent factors include the composition of the naive TCR repertoire (Jenkins and Moon, 2012; Kim et al., 2005; Moon et al., 2007), genetic polymorphisms (i.e., in T cell signaling, T cell–APC interactions, antigen processing genes, and host factors regulating viral replication), and prior exposure to crossreactive pathogens that may influence HIV-1–specific memory T cell frequencies (Campion et al., 2014; Su et al., 2013).

We have previously developed a reductionist cell-free antigen processing system that mimics the MIIC and yields immunodominant epitopes from full-length proteins that induce memory CD4^+^ T cell responses. The system is composed of soluble MHC-II (HLA-DR1*01:01, or DR1), DM, and three cathepsins in an acidic and reducing environment (Hartman et al., 2010; Kim et al., 2014), and as such, uniquely isolates structural and antigenic factors involved in epitope selection. Protein antigens are denatured in this environment, captured by DR1, further selected with the help of DM, and trimmed by cathepsins. DR1-bound peptides are then eluted and sequenced by liquid chromatography tandem mass spectrometry (LC-MS/MS). This system successfully identified the dominant epitopes of several pathogens and autoantigens in mice and humans (Hartman et al., 2010; Kim et al., 2014, 2017). We reasoned that such a system may reveal patterns of epitope selection for HIV-1, a pathogen for which most of our understanding of this process has derived from CD4^+^ T cell responses to overlapping peptides (Lindqvist et al., 2012; Ranasinghe et al., 2012; Soghoian et al., 2012; Laher et al., 2017). Analysis of antigen-dependent factors leading to epitope selection across the HIV-1 proteome, including epitopes associated with viral control, is lacking. Thus, we used our cell-free processing system to understand how DM resistance and antigen structure influence epitope presentation from HIV-1 protein antigens. We hypothesized that our system could provide a broader landscape of potential HIV-1 epitopes and may identify novel epitopes not previously documented in studies of CD4^+^ T cell responses to HIV-1. Vaccine-induced responses to these novel epitopes might contribute to vaccine efficacy even if the relevant epitopes are not normally selected for in infected individuals.

## Results

### Cell-free processing of HIV-1 proteome identifies MHC-II epitopes

**Figure 1.**
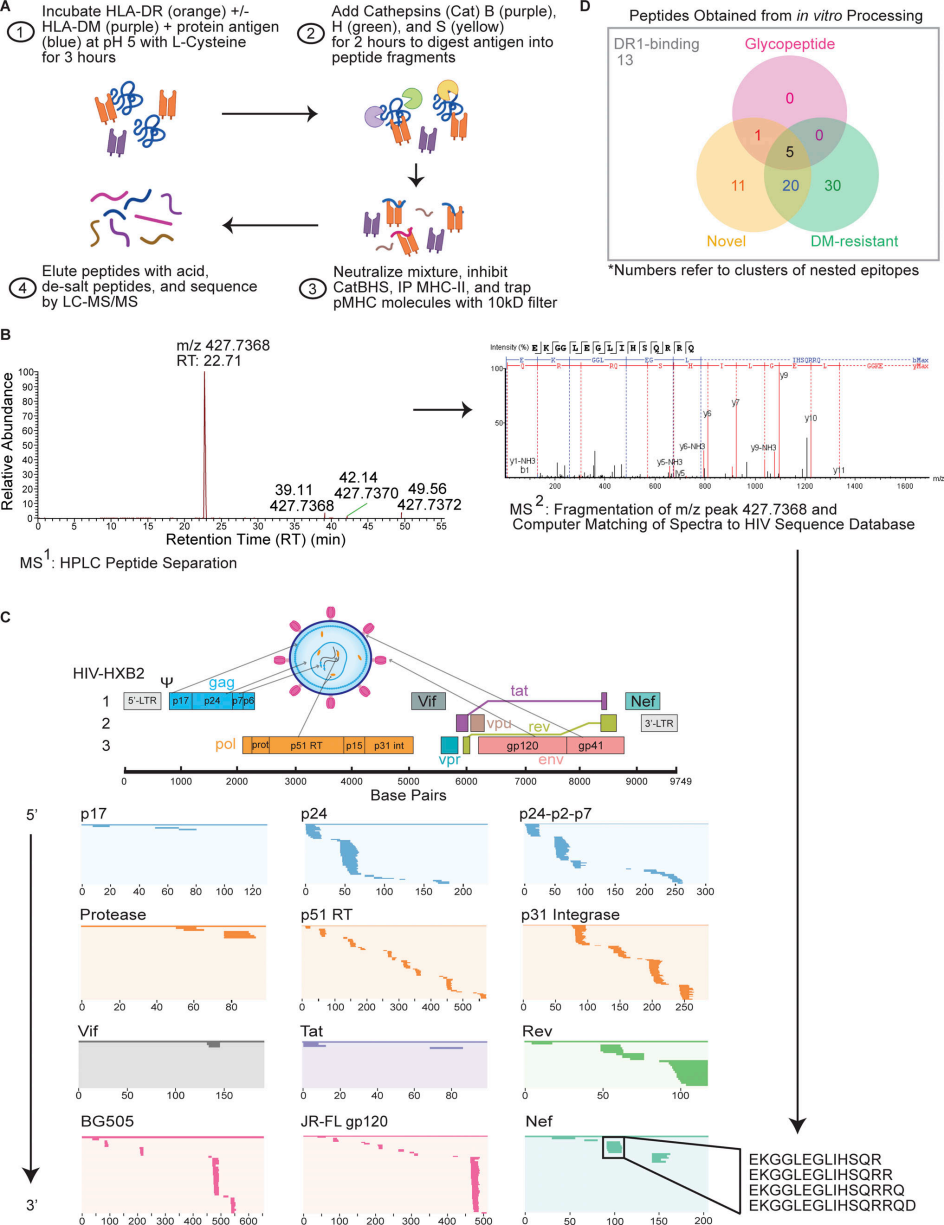
**Cell-free antigen processing system identifies immunodominant epitopes in HIV-1 proteome. (A)** Cell-free processing workflow. HIV-1 protein antigens were incubated with HLA-DR and +/− DM in reducing conditions and low pH for 3 h before the addition of cathepsins B, H, and S (CatBHS) for 2 h. The solution was neutralized, cathepsins inhibited, and DR1 was immunoprecipitated. Peptides were eluted and sequences identified by LC-MS/MS. **(B)** Extracted base peak chromatograph representing a peptide from LC-MS (MS^1^) of Nef cell-free-derived epitopes. Further fragmentation of the peptide (MS^2^) resulted in individual b and y ions corresponding to amino acids for the Nef-EKG_93-108_ epitope (Table S4). **(C)** The HIV-1 genome with its three reading frames is shown, along with the near-full proteome that was subjected to cell-free processing. Locations of epitopes from in vitro processing in all conditions (+/− DM) are shown as lines within the overall protein sequence and listed in 5′ to 3′ order. Shown for Nef within the black box is a cluster of epitopes corresponding to Nef-EKG_93-108_, a hot spot obtained from cell-free processing. **(D)** Venn diagram showing number of epitope clusters identified through cell-free processing of the HIV-1 proteome. Novel epitopes exclude those previously reported to induce memory responses in HIV^+^ individuals or vaccine recipients (LANL, 2018) and have <60% overlap with a literature epitope (Table S4). “13” indicates the number of epitope clusters that bind to DR1 only in the absence of DM and are not novel epitopes or glycopeptides. Data in C and D represent two independent experiments performed per antigen tested in single determinations due to antigen availability and the quantity required per assay.

**Figure S1.**
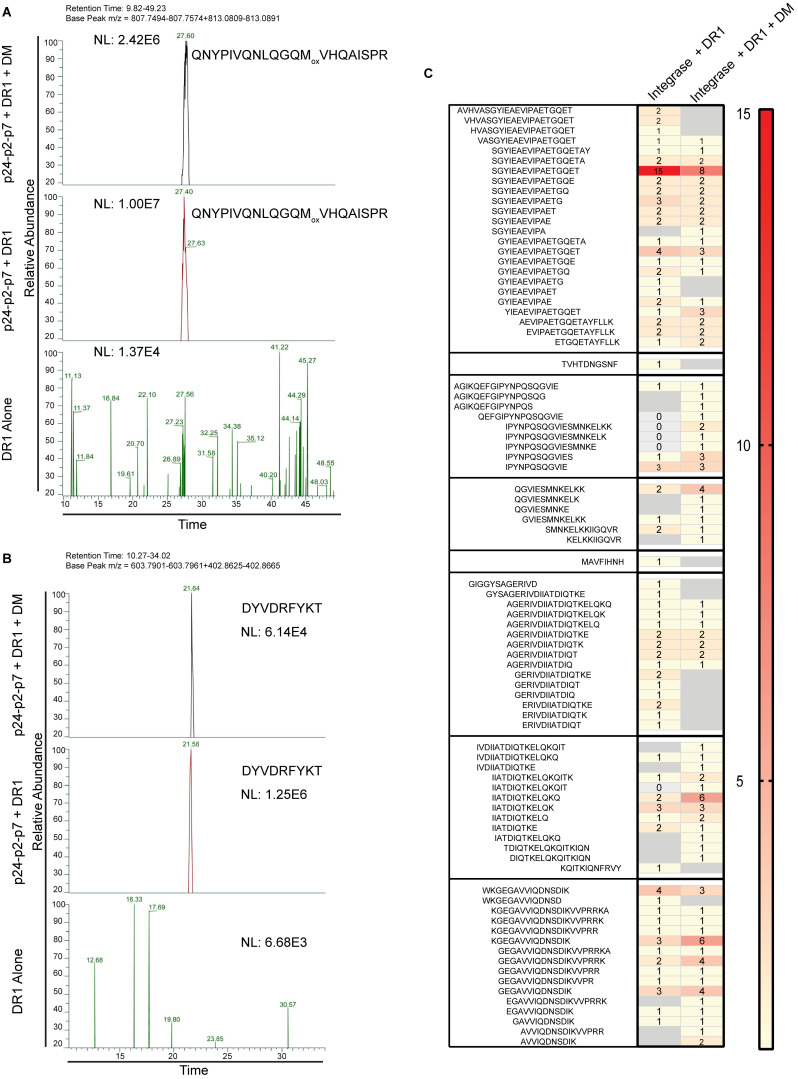
A cell-free processing system utilizing HLA-DR1 (DRB1*01:01), DM, and three cathepsins (B, H, and S) yields DM-sensitive and DM-resistant epitopes that are not observed in a no-antigen control. (A) Extracted base peak chromatographs from p24-p2-p7 from in vitro processing + DM (top panel), −DM (middle panel), or without antigen (bottom panel) highlighting the epitope Gag-QNYPIVQNLQGQM_ox_VHQAISPR. This epitope was identified with oxidized/unoxidized Methionine forms. Neutral loss (NL, measure of peak intensity) at the retention time and m/z for this epitope was 2–3 logs higher (10^6^/10^7^) compared with the no-antigen control (NL ∼ 10^4^), which had expected levels of background noise. **(B)** Extracted base peak chromatograph of p24-p2-p7 highlighting an example of a DM-sensitive epitope, Gag-DYV_295-305_. This epitope was identified with a high NL score without DM (middle) translating to 0 PSMs but lower NL score in the presence of DM (top). Both NL scores are logs higher than the no protein control. **(C)** Representative heat map showing PSM differences for cell-free processing of INT in the presence of DR1 +/− DM. Data in A–C are representative of two independent experiments performed per antigen tested in single determinations.

HIV-1 proteins, as well as individual protein subunits derived from HIV-1 polyproteins, were incubated in a low pH (5.0–5.2) reducing environment with DR1 with or without DM and then digested with cathepsins B, H, and S, followed by MS analysis (Fig. 1, A and B). This protocol mimics the natural conditions and sequence of events in MHC-II antigen processing (Hartman et al., 2010; Kim et al., 2014; Sadegh-Nasseri and Kim, 2015). We specifically allowed for antigen binding to DR1 first before adding cathepsins as this model is facilitated by the open-ended groove of MHC-II and supported by various studies (Castellino et al., 1998; Mimura et al., 2007; Nelson et al., 1997), including those showing that full-length reduced versions of antigens can bind to MHC-II molecules (Kim et al., 2014; Runnels et al., 1997; Sette et al., 1989) and that epitopes from viral proteins are susceptible to cathepsin digestion if not captured by DR1 (Kim et al., 2014). We performed cell-free processing experiments with and without DM, as resistance of a pMHC-II complex to DM-mediated dissociation predicts immunodominance for pathogen-derived proteins better than affinity of the peptide for the MHC or the intrinsic half-life of the complex (Hartman et al., 2010; Yin et al., 2012; Fig. 1, A and B). Epitopes identified in the presence of DM were considered DM-resistant and likely immunogenic (Fig. S1, A and B).

We subjected nearly the entire HIV-1 proteome to cell-free processing (Fig. 1 C and Table S1). Where possible, we performed processing of HIV-1 polyproteins and their individual subunits as various forms of an HIV-1 protein may be present in an infected CD4^+^ T cell whose contents are captured by an APC (Addison et al., 2022). The HIV-1 Gag polyprotein (Pr55^Gag^) is cleaved by the viral protease to liberate the structural proteins matrix (MA/p17), capsid (CA/p24), and nucleocapsid (NC/p7) as well as the unstructured proteins spacer peptide 1 (SP1/p2), spacer peptide 2 (SP2/p1), and p6 (Fig. 1 C; Freed, 2015). We utilized the cell-free processing system on multiple Gag forms: p17, p24, and p24-p2-p7 precursor proteins (Table S1). We also examined the individual HIV-1 enzymes protease (PR), reverse transcriptase (RT), and integrase (INT), which are encoded by the *pol* gene. Despite their lower abundance relative to Gag in infected cells, their critical role in the viral life cycle and high conservation provide a strong impetus to identify immunogenic MHC-II Pol epitopes (Fig. 1 C). Additionally, we subjected monomeric gp120 (JR-FL strain) and a trimeric form of gp140, the extracellular portion of the Env protein (BG505 SOSIP.664; Sanders et al., 2013), to cell-free processing (Fig. 1 C and Table S1). Finally, we analyzed cell-free processing of all accessory proteins available commercially (Vif, Tat, Rev, and Nef; Fig. 1 C and Table S1).

Across the HIV-1 proteome, we observed clusters of nested epitopes containing overlapping sequences with typical CD4^+^ T cell epitope length variation (Fig. 1 C). From 80 identified clusters that could bind to DR1, 55 were DM-resistant and 35 had not previously been reported (Fig. 1 D). Notably, all HIV-1 proteins subjected to in vitro processing—constituting the majority of the viral proteome—generated epitopes that could bind to a single MHC-II allele, DR1 (Fig. 1 C).

### Cell-free processing reveals hot spots of HIV-1 epitopes

**Figure 2.**
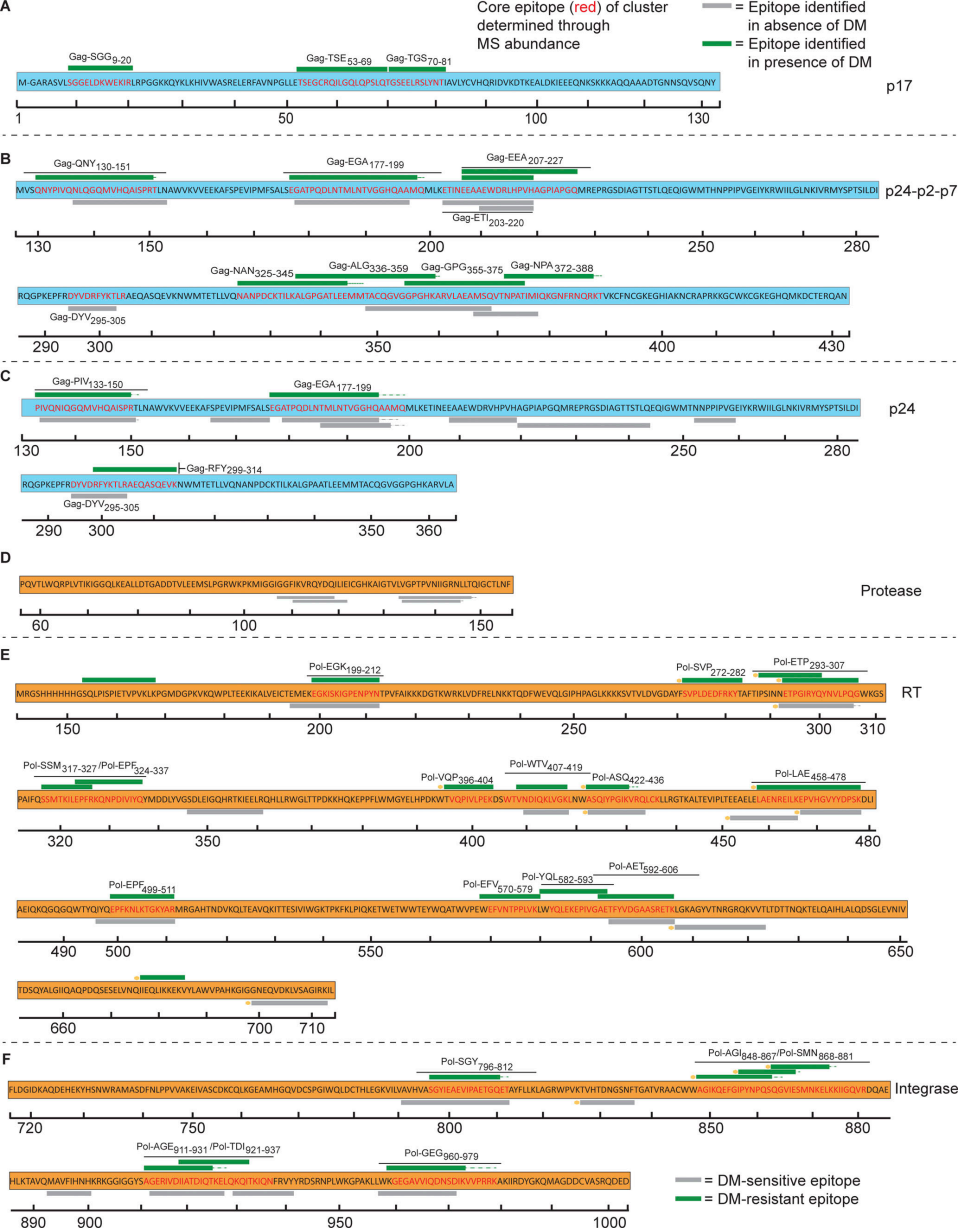
**Cell-free processing of Gag and Pol proteins reveal epitope hot spots.** Epitopes from the HIV-1 Gag and Pol proteins identified by LC-MS/MS from cell-free processing are shown in the form of epitope maps, with epitopes highlighted across the Gag and Pol proteins. **(A–C)** Maps for Gag proteins include (A) p17 (where M- indicates myristoylation of the first Gly residue), (B) p24-p2-p7, and (C) p24. **(D–F)** Epitope maps for Pol proteins include (D) protease, (E) RT, and (F) INT. Green bars indicate epitopes obtained both in the presence and absence of DM (DM-resistant); gray bars indicate epitopes obtained only in the absence of DM (DM-sensitive). Hatched lines indicate additional residues (i.e., “ragged edges”) at the ends of epitopes that were observed (see below, Fig. 4, A and B). For each epitope cluster, the core epitope was defined using the peptide with the greatest number of PSMs (see below, Fig. 4, A and B). Novel epitopes (<60% overlap with existing 2018 LANL Database epitopes, see Table S4) are indicated with gold circles. Epitope maps in A–F represent two independent experiments performed per antigen tested in single determinations.

**Figure 3.**
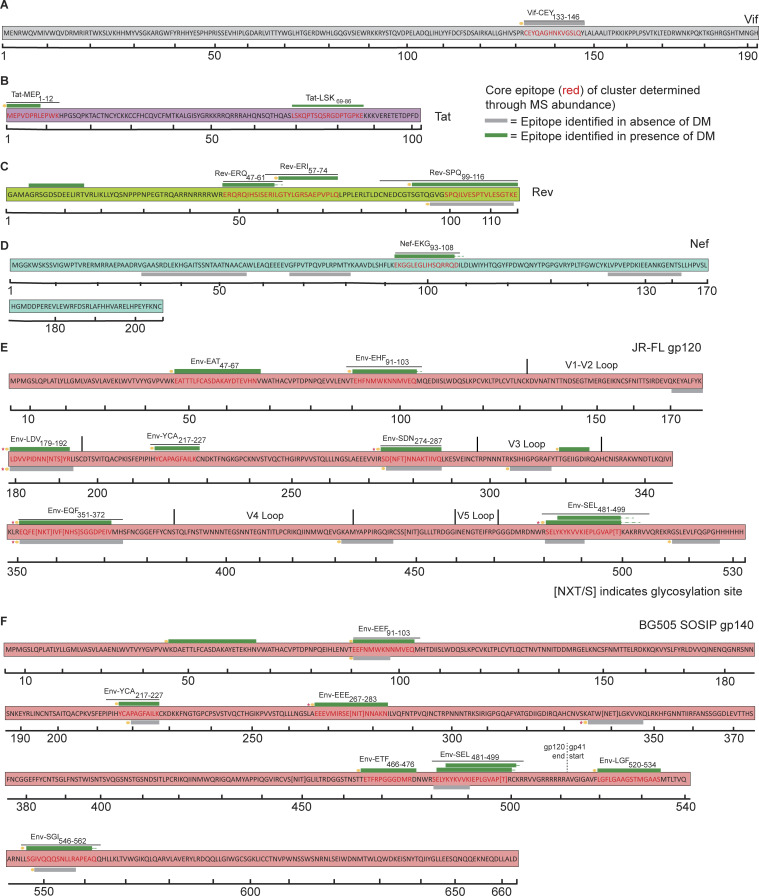
**Cell-free processing of HIV-1 accessory proteins and Env yields epitope hot spots and reveals overlapping epitopes. (A–D)** Epitope maps of (A) Vif, (B) Tat, (C) Rev, and (D) Nef peptides obtained from in vitro cell-free processing. **(E and F)** Epitope maps of peptides identified from cell-free processing of (E) gp120 (JR-FL strain) and (F) gp140 (BG505 SOSIP). As above, green bars indicate epitopes obtained both in the presence and absence of DM (DM-resistant); gray bars indicate epitopes obtained only in the absence of DM (DM-sensitive). Novel epitopes are indicated by gold circles. Pink stars indicate epitopes containing an N- or O-linked glycosyl moiety. Epitope maps in A–F represent two independent experiments performed per antigen tested in single determinations.

Epitope “hot spots” were apparent in all proteins tested (Fig. 1 C). Cell-free processing of the myristoylated matrix protein (Myr-MA) yielded three DM-resistant epitopes (Fig. 2 A), while processing of p24-p2-p7 (Newman et al., 2004) and p24 yielded seven and three clusters of DM-resistant epitopes, respectively (Fig. 1 C and Fig. 2, B and C). Processing of Pol proteins also produced several epitope clusters (Fig. 2, D–F). INT processing revealed nested sets of epitopes (Fig. S1 C) that resided within four discrete locations (Fig. 1 C and Fig. 2 F). Of the accessory proteins Vif, Tat, Rev, and Nef, individual epitope hot spots were also observed (Fig. 1 C and Fig. 3, A–D). Cell-free processing of the transcriptional activator Tat (Rice, 2017) yielded epitopes near the N′ and C′ termini (Fig. 3 B). Rev yielded four DM-resistant epitope clusters, including a predominant epitope cluster of Rev-SPQ_99-116_ from the C-terminus (Fig. 1 C and Fig. 3 C). Cell-free processing of Nef led to four main epitope clusters, with a single DM-resistant epitope (Fig. 1 C and Fig. 3 D). Finally, cell-free processing of gp120 and gp140 proteins from HIV-1 also yielded clear hot spots (Fig. 3, E and F).

**Figure 4.**
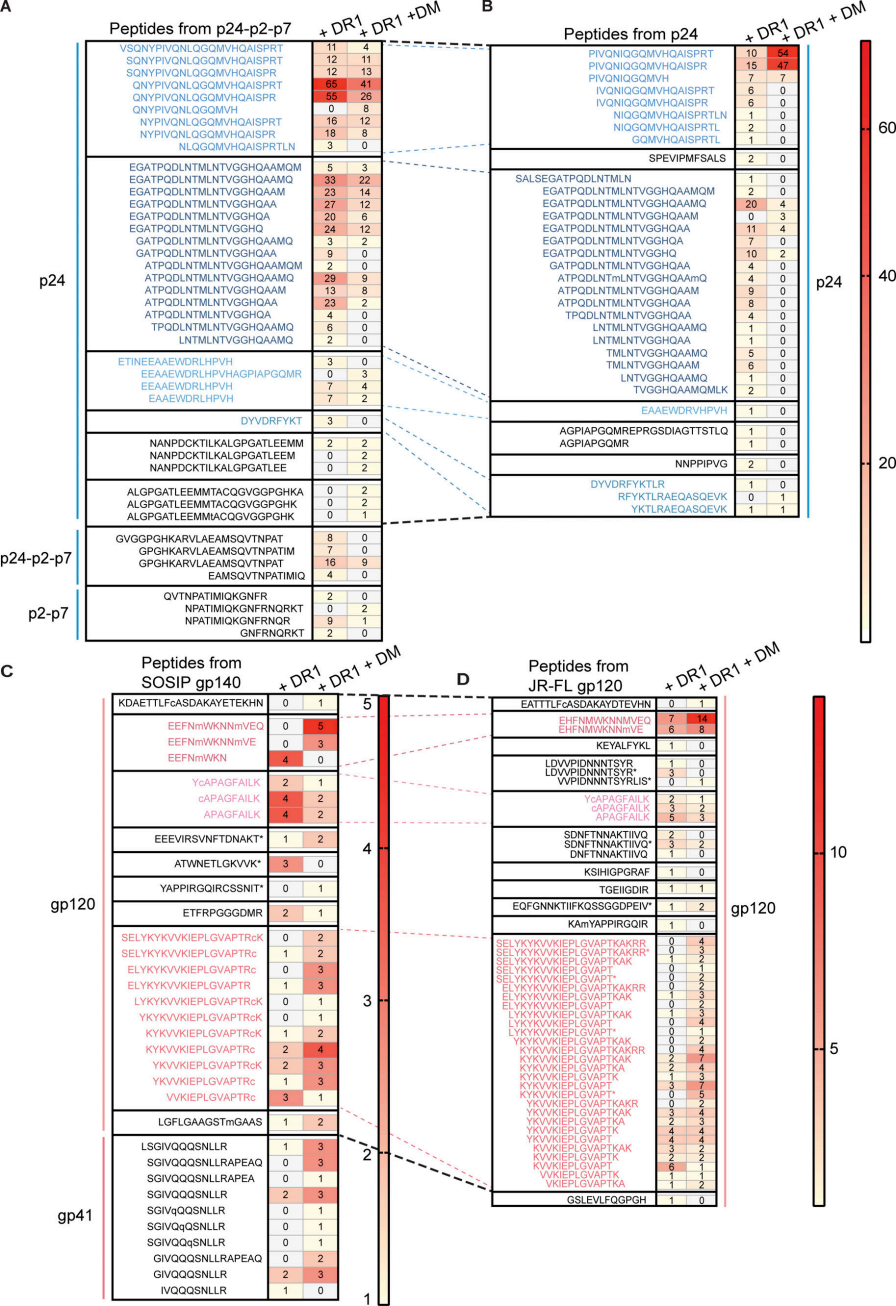
**Cell-free processing reveals similarities in epitopes from HIV-1 polyproteins versus individual subunits. (A and B)** Heat maps displaying PSMs identified at >95% probability from in vitro processing of (A) p24-p2-p7 (eight clusters) or (B) p24 (seven clusters) in the presence of DR1 +/− DM. Peptides within solid lines indicate a cluster or nested set of epitopes. Peptide clusters were defined based on shared start and end residues, as well as the extent of overlap between the P1 and P9 anchor residues for DR1. p24-p2-p7 contains a VSQNY extension from p17 at the 5′ end. Slight sequence differences from the p24 portion of p24-p2-p7 (utilizing NL4.3 lab strain, which contains the NY5 sequence for Gag) compared to p24 alone (HXB2, an infectious molecular clone of LAV) can be observed in certain epitopes, such as the Leu to Ile mutation in Gag-PIV: PIVQNIQGQMVHQAISPR. **(C and D)** Heat maps showing PSM differences from cell-free processing of (C) gp140 (BG505 SOSIP) and (D) gp120 (JR-FL) in the presence of DR1 +/− DM. Black hashed lines in A–D indicate where the individual protein (i.e., p24) derives from the polyprotein (i.e., p24-p2-p7). Light blue (Gag) or pink (Env) hashed lines indicate common epitopes shared between the individual protein (B, D) and the polyprotein (A, C). Asterisks in C and D indicate glycopeptides.

In addition to hot spots, we observed similarities in epitopes identified in HIV-1 polyproteins and individual subunits of those polyproteins, for instance with p24-p2-p7 and p24. Peptide spectral matches from MS data provided information on relative abundance of peptides within the sample (Zybailov et al., 2005). For both p24-p2-p7 and p24, two of the most abundant epitopes from cell-free processing were the overlapping epitope cluster Gag-QNY_130-151_/Gag-PIV_133-150_ (Fig. 2, B and C) as well as Gag-EGA_177-199_ (Fig. 4, A and B). This pattern of shared epitope selection between individual subunits and polyproteins was also observed with our analysis of gp140 and gp120. For both gp140 and gp120, the three epitopes with the greatest abundance were Env-EHF_91-103_, Env-YCA_217-227_, and Env-SEL_481-499_ (Fig. 4, C and D). In vitro processing of gp120 from a different HIV-1 isolate (LAV) yielded the same three dominant epitopes (data not shown). The finding that gp120 and trimeric gp140 yielded overlapping epitopes suggests that the denaturing environment of the MIIC sufficiently exposes most gp120/gp140 high-affinity DR1 binding sites regardless of original tertiary structure and allows the trimer form to behave similarly to destabilized monomers.

The location of epitopes in certain hot spots suggested a structural etiology. To understand whether structural features of a protein antigen led to epitope selection from in vitro processing (Landry, 1997), we analyzed the solvent-accessible surface area (ASA) and thermal stability of the HIV-1 protein antigens as inferred from crystal structures (Mettu et al., 2016). Protein thermal instability is likely critical for epitope dominance: unstable regions unfold first and become available to antigen-processing machinery, leading to increased abundance of certain epitopes. To examine protein folding stability (hereafter referred to as simply protein stability), we used a thermodynamic analysis of the ensemble of possible partially unfolded states (the COREX/BEST [Biology using Ensemble-based Structural Thermodynamics] algorithm), which has been validated by Hydrogen/Deuterium exchange (Hilser and Freire, 1996; Hilser et al., 1998, 2006; Pan et al., 2000; Whitten et al., 2005) as well as nuclear magnetic resonance (NMR)–monitored acid denaturation and cold denaturation of proteins (Babu et al., 2004; Hilser and Freire, 1996; Liu et al., 2012; Whitten et al., 2006; see Materials and methods). Using this algorithm, we could predict which regions of a protein structure are less stable and more likely to unfold.

### Structurally unstable regions of HIV-1 proteins predict epitope dominance

**Figure 5.**
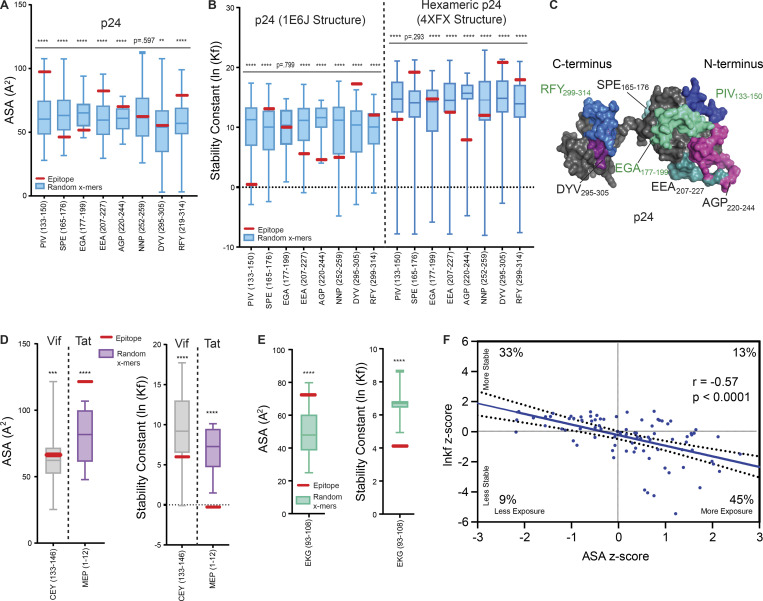
**Exposed or structurally unstable regions of HIV-1 proteins predict epitope dominance. (A)** Epitopes from p24 (Fig. 4 B) were analyzed for solvent accessibility (ASA) by PDB PISA algorithms from the PDB structure 1E6J. Higher values of ASA indicate higher epitope exposure. Data are displayed as box-and-whisker plots showing the epitope of interest (EOI) in red compared to a distribution of average ASA values of random x-mers spanning the entirety of the protein (derived via sliding scale analysis, see Materials and methods). These distributions exclude the EOI in red so that its stability relationship with the rest of the protein can be visualized. **(B)** Epitopes from p24 were analyzed for stability, expressed as average stability constants (lnKf) by the COREX/BEST algorithm, from PDB structures 1E6J and 4XFX. Data are displayed in box-and-whiskers plots as in A. Lower values of lnKf indicate lower epitope stability. **(C)** p24 epitopes obtained from cell-free processing in the absence (black text labels) or presence (green text labels) of DM are highlighted (PDB: 1E6J). Epitope NNP_252-259_ located on the posterior surface of 1E6J is not shown. **(D and E)** Comparison of accessibility (PDB PISA, left) and stability (COREX, right) of dominant epitopes obtained from cell-free processing of Vif (D, gray), Tat (D, purple), and Nef (E) relative to a distribution of randomly generated x-mer epitopes spanning these proteins (see Methods), excluding the EOI. EOI is shown in red. Structures used for analysis in D and E are shown in Table S1. **(F)** Two-tailed Pearson correlation was used to analyze the relationship between z-scores of ASA (PDB PISA) versus lnKf (stability, COREX) for all peptides obtained from cell-free processing. Best fit line is shown encased in a 95% confidence interval. Quadrants dividing the data into more/less stable and more/less accessible regions of the graph were used to obtain frequencies of epitopes within each group. Data in A, B, D, and E that were normally distributed were subject to a one-sample, two-tailed *t* test, and non-normally distributed data were subject to a two-tailed Wilcoxon Signed Rank Test, comparing the mean (*t* test) or median (Wilcoxon Signed Rank) of the distribution to the mean stability of the epitope. **P < 0.01; ***P < 0.001; ****P < or = 0.0001.

**Figure S2.**
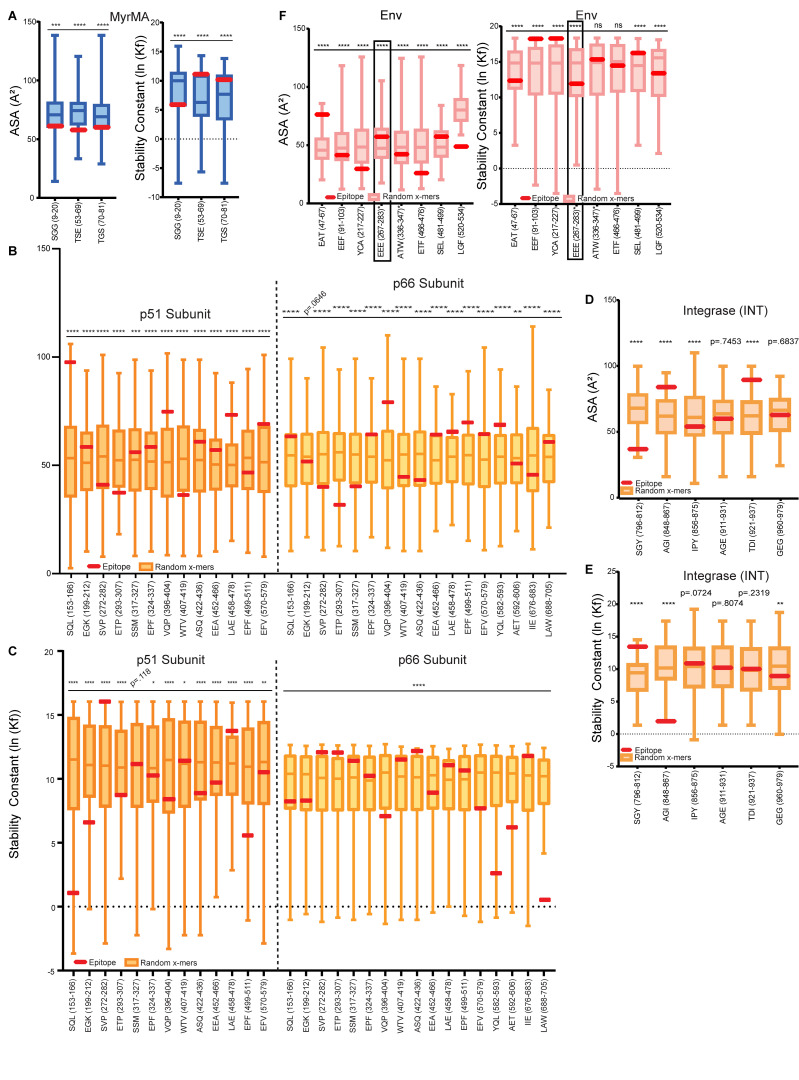
**Accessibility and stability trends of HIV-1 epitopes from cell-free antigen processing. (A)** Epitopes from Myr-MA were analyzed by PDB PISA and COREX/BEST algorithms. Box-and-whiskers plots show distribution of average solvent ASA (left) or stability constants (right) of random x-mers spanning the protein (derived via sliding scale analysis, see Materials and methods). These distributions exclude the EOI, shown in red so that its accessibility/stability relationship to the rest of the protein can be visualized. In these plots, higher ASA values indicate higher epitope exposure and lower lnkf values indicated decreased epitope stability. **(B and C)** Box-and-whiskers plots showing the distribution of (B) average ASA or (C) average stability constants from randomly generated p51 and p66 epitopes excluding the EOI. EOI in red. Stability constants were determined using COREX based on the 1HMV structure of RT, analyzing each subunit independently. **(D and E)** Box-and-whiskers plots showing the distribution of average (D) ASA or (E) stability of random x-mer epitopes spanning the INT protein (1EX4) and excluding the EOI. **(F)** Box-and-whiskers plot showing the distribution of average ASA (PDB PISA; left) or stability (COREX; right) of random x-mer epitopes spanning the BG505 SOSIP protein (4ZMJ) and excluding the EOI, with a box highlighting the EEE glycopeptide. EOI is shown in red. Normally distributed data in A–F were subject to a one-sample, two-tailed *t* test, and non-normally distributed data were subject to a two-tailed Wilcoxon Signed Rank Test, comparing the mean (*t* test) or median (Wilcoxon Signed Rank) of the distribution to the mean ASA or stability of the epitope. *P < 0.05; **P < 0.01; ***P < 0.001; ****P < or = 0.0001.

Structural analysis was performed for HIV-1 protein antigens with available crystal structures (Table S1). For small, monomeric proteins for which the COREX algorithm was designed, epitopes obtained by cell-free processing were located in regions of low stability (Fig. 5 and Fig. S2, A–F). For example, the abundant Gag-QNY_130-151_/Gag-PIV_133-150_ epitope is contained within the β-hairpin loop at the N-terminus of p24 (Cortines et al., 2011). Protein termini may be more available to bind to DR1 for subsequent processing. Indeed, Gag-PIV_133-150_ contained significantly higher accessibility and lower stability than the rest of the protein (Fig. 5, A and B). Notably, only the C-terminal portion of the Gag-PIV_133-150_ epitope was resolved from the 1E6J structure, which may reflect this region’s more dynamic nature (Fig. 5 C). A different p24 structure containing the full epitope sequence provided the same conclusions (Fig. 5 B, right). Thus, structural features of Gag-PIV_133-150_ may promote its presentation.

Structural analysis also provided insights on epitope locations within HIV-1 accessory proteins obtained from cell-free processing. Processing of Vif yielded the DR1-binding epitope Vif-CEY_133-146_ (Fig. 3 A), which had high accessibility and low stability (Fig. 5 D). Processing of the transcriptional activator Tat (Rice, 2017) also revealed a novel N-terminal epitope Tat-MEP_1-12_, which had high accessibility and low stability (Fig. 5 D). Cell-free processing of Nef in the presence of DM led to convergence on a single epitope (Nef-EKG_93-108_; Fig. 3 D), also from a region with high accessibility and low stability (Fig. 5 E). The most abundant epitope from cell-free processing of Rev, C-terminal Rev-SPQ_99-116_ (Fig. 3 C), was in a region absent from the Protein Data Bank (PDB) structure, potentially reflecting a more disordered conformation. Altogether, in vitro processing of accessory proteins generally led to selection of epitopes with high accessibility and low stability.

COREX was less predictive of stability patterns for larger proteins, such as RT, a heterodimer comprised of a 66 kD subunit (p66) and 51 kD subunit (p51), and gp140 (Fig. S2, B–E). 60% of epitopes from RT were solvent accessible and 56.7% had low stability constants (Fig. S2, B and C). However, when all epitopes from cell-free processing were analyzed in aggregate, two-thirds of the epitopes obtained were located in regions of lower stability or higher accessibility (Fig. 5 F), and there was a statistically significant inverse correlation between accessibility and stability (r = −0.57, P < 0.0001). Thus, dominant epitopes identified by cell-free processing were associated with regions of lower stability and higher accessibility, with almost half of the epitopes having both these characteristics.

### DM influences epitope diversity and relative abundance

**Figure 6.**
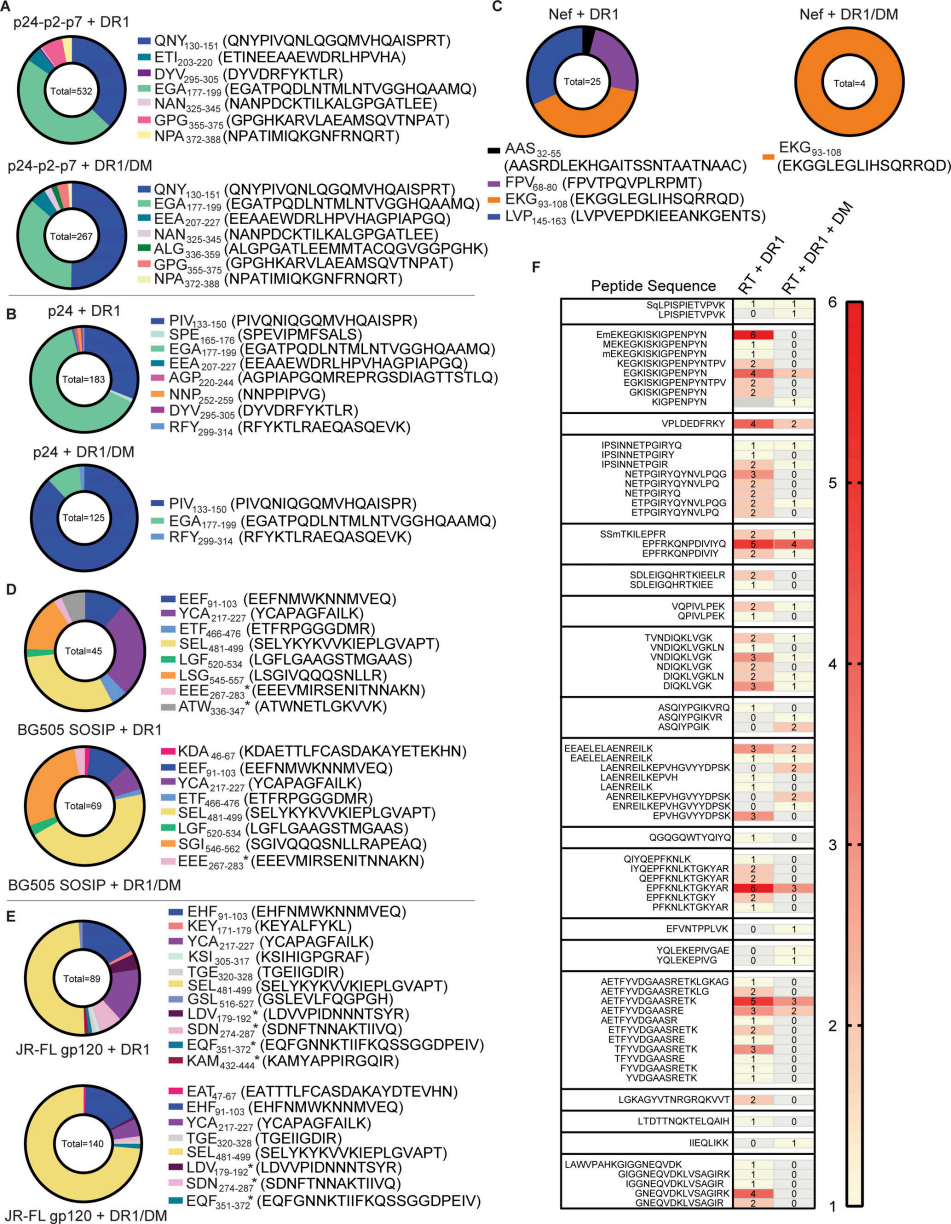
**Cell-free processing displays influence of DM influences on epitope dominance and relative abundance. (A and B)** Relative differences in abundance of various (A) p24-p2-p7 or (B) p24 epitopes are shown as differences in PSMs in the absence (above) or presence (bottom) of DM. The total number of PSMs within pie charts was obtained by summing all PSMs for the protein antigen (see Materials and methods for peptide identification criteria). **(C)** Relative differences in abundance of Nef epitopes are shown as differences in PSMs in the absence (left) or presence (right) of DM, with convergence on one dominant epitope (EKG_93-108_) in the presence of DM. **(D and E)** Relative differences in PSMs from cell-free processing of (D) gp140 (BG505) or (E) gp120 (JR-FL) are shown in the absence (top) or presence (bottom) of DM. **(F)** Peptide sequences from in vitro processing of RT fell into 19 major clusters. Data are shown as a heat map with PSMs in the presence of DR ± DM. 14 clusters are observed in the presence of DM. Data in A–F represent two independent experiments performed per antigen tested in single determinations.

As stated above, cell-free processing experiments were performed with and without DM, as resistance to DM-mediated dissociation predicts epitope dominance (Hartman et al., 2010; Yin et al., 2012; Fig. 1, A and B). Indeed, we found that the inclusion of DM narrowed the diversity of peptides identified from cell-free processing, in some cases to one epitope. In vitro processing of p24-p2-p7 in the presence of DM reduced the number of peptide spectra from 532 to 267 sequences and revealed Gag-QNY_130-151_/Gag-PIV_133-150_ as the most abundant epitope cluster (Fig. 6 A). For in vitro processing of p24 alone (without competition from p2 and p7), Gag-PIV_133-150_ and Gag-EGA_177-199_ again dominated peptide spectra (Fig. 6 B). In this case, there was an even more dramatic shift in abundance from the Gag-EGA to the Gag-QNY/PIV epitope cluster in the presence of DM. The ability of the Gag-QNY/PIV epitope cluster to withstand DM-mediated dissociation reflects the sequence of QNYPIVQNLQGQMVQAISPRT and the biochemical nature of DM resistance, which requires an epitope to contain a large hydrophobic residue to fill the P1 pocket of DR1 (Chou and Sadegh-Nasseri, 2000; Stern et al., 1994). The Ile, Val, and Leu contained within Gag-QNY/PIV are candidate P1 pocket residues. Importantly, the core epitope required for binding to DR1 (QNYPIVQNLQGQMVQAISPRT; the underlined portion of the epitope corresponds to the minimal core epitope required to bind to the peptide-binding groove of DR1, as determined by Harcourt et al., 1998) was contained within nearly all DM-resistant peptides from the Gag-QNY_130-151_/Gag-PIV_133-150_ cluster (Fig. 4, A and B), and PIVQNLGQMVHQAISPRL and QGQMVQAISPRTLN bind with high affinity to DR1 (Harcourt et al., 1998; Wilson et al., 2001). Thus, the peptide sequence, high affinity for DR1, DM resistance of Gag-QNY_130-151_/Gag-PIV_133-150_, and the location of the epitope in a lower stability region as discussed above, may promote its presentation.

Remarkably, the addition of DM to in vitro processing of Nef also narrowed the peptide repertoire, in this case to a single epitope (Nef-EKG_93-108_; Fig. 6 C). This epitope has been shown to induce CD4^+^ T cell responses in HIV^+^ individuals (Table S2). In another example, DM narrowed the diversity of peptides obtained from cell-free processing of gp140 and gp120: the epitope Env-SEL_481-499_ represented 50 and 75% of DM-resistant peptides derived from cell-free processing of gp140 (Fig. 6 D) and gp120 (Fig. 6 E), respectively. This peptide is also a known CD4^+^ T cell epitope (Table S2) and binds to DR1 with an IC_50_ of 4 nM (Fonseca et al., 2006; Fig. 4 D and Fig. 6 D, top). Its MS abundance may reflect an optimal DR1 core sequence (YKVVKIEPL) that would favor capture of the antigen by DR1 and relatively higher accessibility (Fig. S2 F), but not necessarily lower stability. This could be a case where optimal sequences override less-than-optimal structures. In another example highlighting the role of DM in influencing epitope selection, three DM-resistant epitopes from integrase were most abundant by MS (Pol-SSM_317-327_/Pol-EPF_324-337_, Pol-AET_592-606_, and Pol-WTV_407-419_; Fig. S1 C); these epitopes have previously been shown to elicit CD4^+^ T cell responses from people living with HIV (PLWH; Table S2).

Unexpectedly, in some instances, in vitro processing produced only DM-sensitive epitopes. This was the case for PR and Vif (Fig. 2 D and Fig. 3 A). This finding may reflect fewer optimal DR1-binding registers within these relatively smaller protein sequences. In other cases, DM did not appear to narrow peptide species diversity. Cell-free processing of RT produced 19 epitope clusters (Fig. 6 F). Most clusters contained at least one DM-resistant peptide, and several DM-resistant epitopes that are more abundant by MS (Pol-SSM_317-327_/Pol-EPF_324-337_, Pol-AET_592-606_, and Pol-WTV_407-419_) have previously been shown to elicit CD4^+^ T cell responses from PLWH (Table S2). Notably, two DM-resistant epitopes identified in our assay (Pol-ETP_293-307_ and Pol-EEA_452-466_/Pol-LAE_458-478_) are novel (Table S2). Given the high numbers of DM-resistant DR1-restricted RT epitopes observed, it is surprising that more RT-specific CD4^+^ T cell responses have not been documented. This may reflect low levels of RT expression or an antigen-independent factor (such as a lower frequency of naive T cells recognizing Pol proteins; Campion et al., 2014).

**Figure S3.**
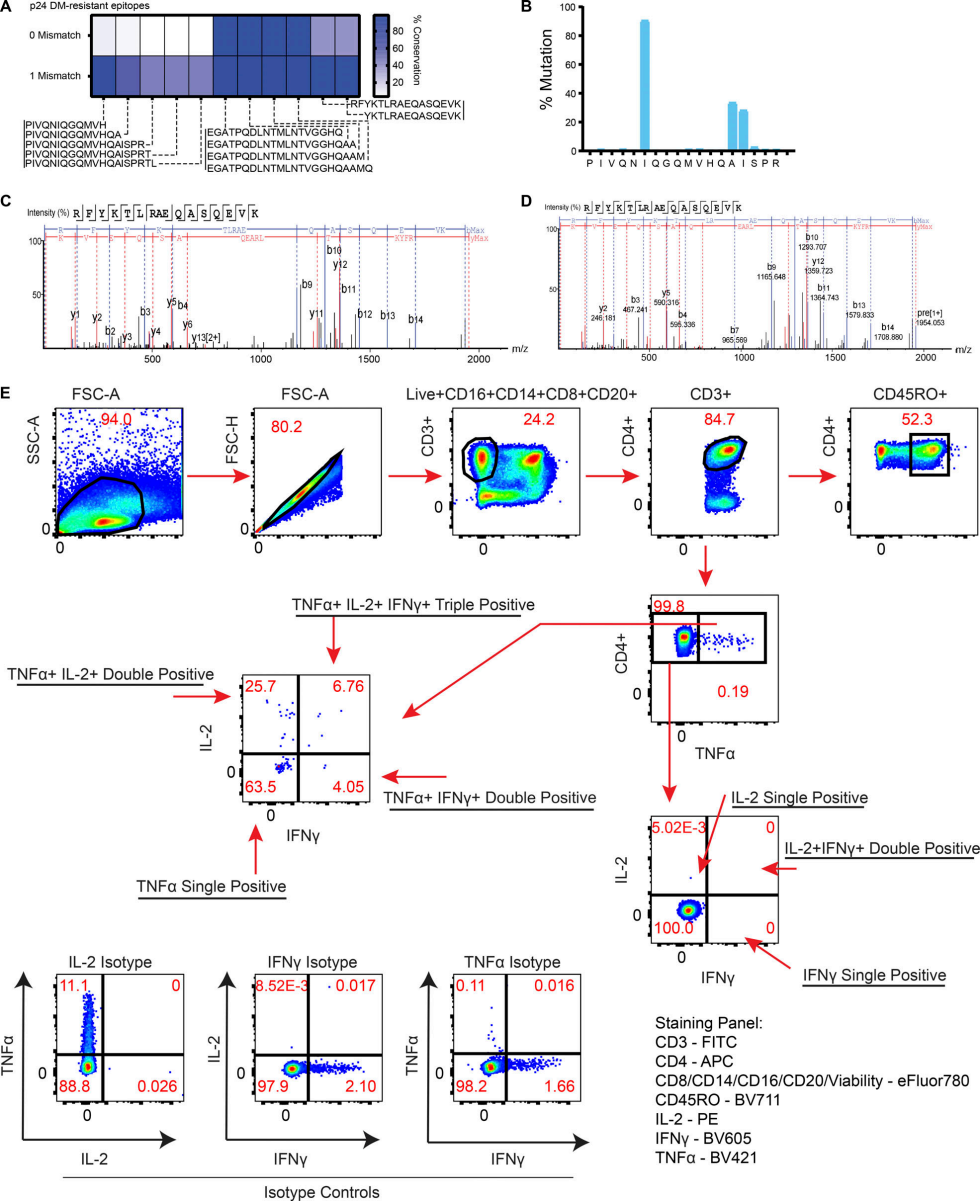
**Mutational and peptide detection characteristics of cell-free-derived HIV-1 epitopes, and gating scheme for measuring CD4**^**+**^ **T cell responses from PLWH. (A)** Heat map showing epitope sequence prevalence of all epitopes obtained from cell-free processing of p24 compared with 1066 Clade B sequences (LANL), with either 0 or 1 aa mismatch tolerated. **(B)** Percentage of mismatches per residue is shown for Gag-PIV_133-150_ from p24-HXB2. **(C)** MS/MS scan shows b/y ions for a low-abundance, DM-resistant epitope RFYKTLRAEQASQEVK (Gag-RFY_299-314_) from p24 cell-free processing identified with 1 PSM. **(D)** MS/MS scan of same epitope (Gag-RFY_299-314_), this time synthesized and spiked in at 25 femtomole quantity into murine B cell peptide elution samples containing self-peptides. **(E)** Representative gating strategy for single-, double-, and triple-positive intracellular cytokine responses gated off of Live^+^CD3^+^CD4^+^ T after 16 h of PBMC incubation with single HIV-1 peptides from cell-free processing. Fluorescence minus one controls containing the relevant isotype antibodies were used for gating for cytokines, and CD45RO^+^ was used to confirm that responses were in the memory compartment.

Overall, these results show that while the extent of DM-mediated dissociation can differ for different protein antigens, the presence of DM in the antigen-processing compartment clearly affects epitope abundance, and that epitope hierarchy is first governed at the level of antigen processing (Sadegh-Nasseri and Kim, 2019). More abundantly presented epitopes may induce stronger T cell responses, leading to immune escape. Indeed, the abundant Gag-PIV_133-150_ epitope discussed above was among the least conserved of p24 epitopes (Fig. S3 A), with observed mutations affecting the Ile, Ala, and Ile residues (Fig. S3 B) that may affect TCR recognition rather than binding to DR1 (Harcourt et al., 1998). Thus, the resistance of an epitope to DM-mediated dissociation and the expected increase in epitope density on the cell surface may influence the number of T cells responding to the presented pMHC-II.

### Cell-free system exposes inefficiency in processing of protective epitopes

Integrating protein structure and DM resistance provided insights into a surprising observation: the low abundance of the C-terminal Gag-RFY_299-314_ (RFYKTLRAEQASQEV) epitope from cell-free processing (Fig. 6, A and B). This epitope largely overlaps a previously described, highly conserved immunodominant epitope Gag293 (FRDYVDRFYKTLRAEQASQE), which elicits responses in >50% of PLWH (Kaufmann et al., 2004) and is associated with control of viral replication (Ranasinghe et al., 2012; Vingert et al., 2010). Unexpectedly, we observed few peptide spectral matches (PSMs) in this region of Gag: one PSM for Gag-DYV_295-305_ (DYDRFYKT) from p24-p2-p7 that was DM-sensitive (Fig. 4 A) and two PSMs from p24 alone (RFYKTLRAEQASQEV and YKTLRAEQASQEV) that were DM-resistant (Fig. 4 B). We did not observe these latter two peptides from p24-p2-p7 (Fig. 4 A), possibly reflecting competition from p2/p7 epitopes and/or the different conformation of the polyprotein. The low PSM number is unlikely to be due to our detection limit as we detected femtomole quantities of these peptides in spiked-in experiments (Fig. S3, C and D). As YVDRFYKTLRAEQASQEV had an experimental IC_50_ value of 5 nM for binding to DR1 (Ranasinghe et al., 2013), poor peptide binding to DR1 is unlikely to be responsible. We conclude that although Gag-RFY_299-314_ is immunodominant from the perspective of the T cell response, it is processed inefficiently from p24. Gag-RFY_299-314_ is highly accessible (Fig. 5 A) but located in a highly stable protein region, in contrast to most p24 epitopes that are located in regions of low to average stability (Fig. 5 B). Notably, another low-abundance epitope associated with spontaneous control (AFSPEVIPMFSALSEGA; the underlined part of this previously published epitope refers to the specific sequence that was observed by MS from our cell-free analysis; Fig. 4 B; Ranasinghe et al., 2012) was also located in a high-stability region (Fig. 5 B). Overall, p24 appears to have highly conserved epitopes associated with immune control of viral replication that may not be well presented, and improved immunogen design may allow for enhanced protective T cell responses.

### In vitro processing yields epitopes not previously described from T cell response data

Encouragingly, all HIV-1 proteins yielded epitopes via cell-free processing that corresponded to epitopes previously reported to induce memory CD4^+^ T cell responses (Table S2). As hypothesized, however, we found that our cell-free processing system revealed novel epitopes as well, most apparent from Pol, Env, and accessory proteins. We identified two novel DM-resistant epitopes from RT (Pol-ETP_293-307_ and Pol-EEA_452-466_/Pol-LAE_458-478_; Table S2). From INT, the cell-free processing system also yielded two novel epitopes—Pol-AGI_848-867_ and Pol-SMN_868-881_ (Table S2)—which map to regions of high accessibility, low stability, and low mutation frequency, suggesting potential utility in HIV-1 vaccine design.

**Figure 7.**
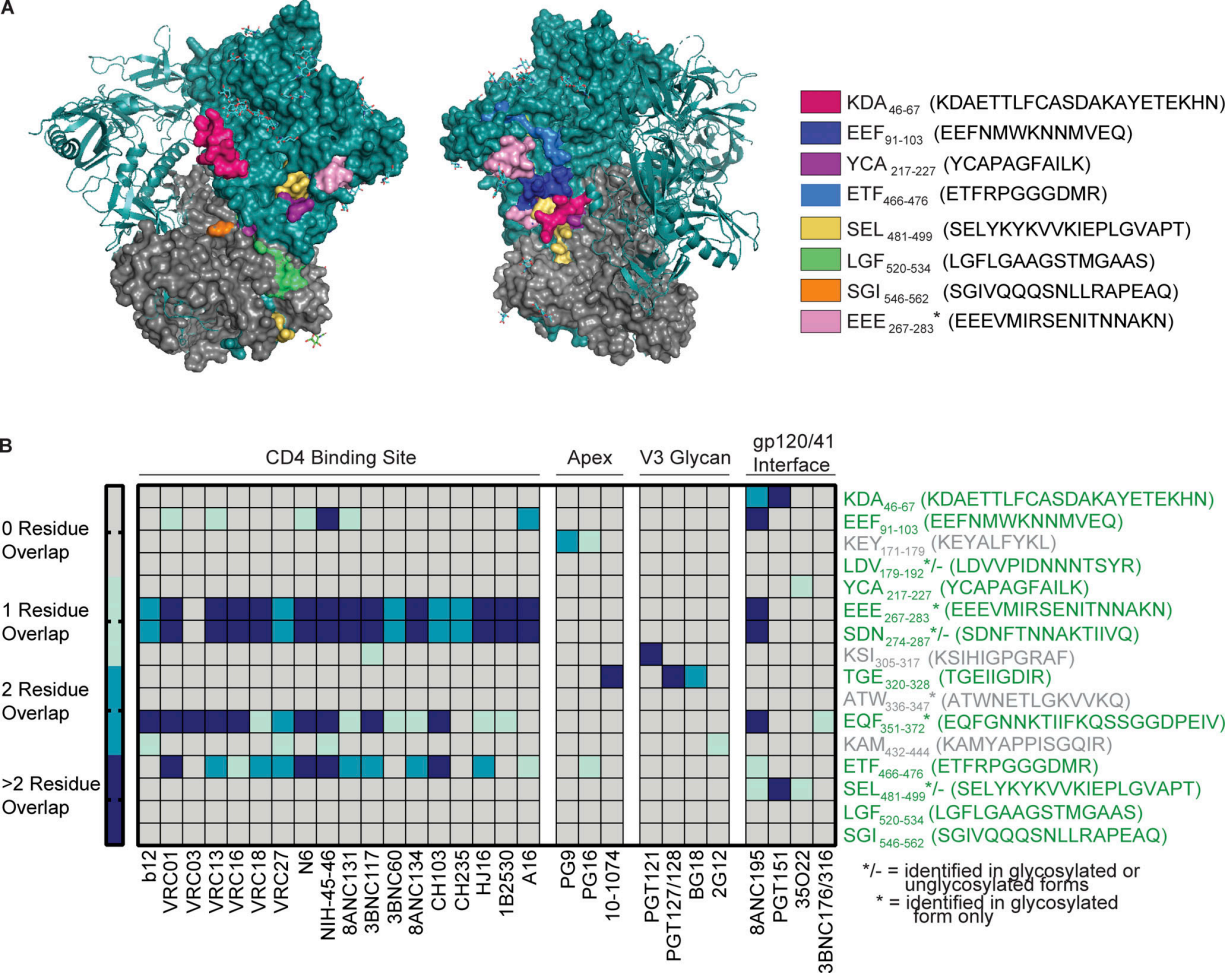
**Most Env epitopes are located in the gp120/41 interface and CD4 binding site. (A)** Visualization of DM-resistant epitopes within the full BG505 trimer (PDB 4ZMJ). gp120 monomers are shown in teal (one monomer as a surface depiction and two monomers as ribbons). Trimeric gp41 ectodomain is shown in gray and glycans in cyan. * indicates EEE_267-283_ is a glycopeptide. **(B)** Epitopes from cell-free processing ± DM of gp120 and gp140 are displayed in a heat map showing their overlap with the binding footprint of several well-characterized bNAbs (HIV LANL Database). */− indicates epitopes identified in glycosylated or unglycosylated forms, while * indicates epitopes identified only in glycosylated form. Epitopes in gray were identified in the absence of DM, while epitopes in green were identified in the presence or absence of DM. Data represent two independent experiments performed per antigen tested in single determinations.

Analyzing the epitopes identified from Env cell-free processing also revealed novel epitopes. Of the three most abundant DM-resistant epitopes (Env-EHF_91-103_, Env-YCA_217-227_, and Env-SEL_481-499_), only Env-SEL_481-499_ had been previously described (Table S2). Processing of the BG505 SOSIP trimer also yielded a novel gp120 epitope, Env-ETF_466-476_, and two novel DM-resistant gp41 epitopes: Env-SGI_546-562_ and Env-LGF_520-534_ (Fig. 4 C), the latter epitope overlapping with the gp41 fusion peptide (Fig. 3 F). In addition to these peptides, we wondered whether we could be missing potential glycosylated epitopes by our analysis. Glycans comprise ∼50% of HIV-1 gp120 by weight (Shen et al., 2014). The gp120/gp140 epitopes revealed by in vitro processing were primarily located in the vicinity of the gp120/gp41 interface or the CD4 binding site (Fig. 7, A and B). Epitopes were not found in variable loops, potentially reflecting the enrichment in N-linked glycosylation sites in these loops (Fig. 3, E and F).

To uncover potential glycosylated epitopes that may be overlooked by traditional LC-MS/MS, we analyzed our MS data on DR1-bound peptides from in vitro processing using GPQuest, an algorithm that evaluates glycopeptides (Sun et al., 2016; Toghi Eshghi et al., 2015). We identified four glycopeptide families from gp120 and two from gp140/BG505 (Table S3), which to our knowledge represent the first HIV-1 glycopeptides that can bind to a human DR molecule. As the gp120 and trimeric gp140 analyzed here were produced in 293T cells, their O- or N-linked glycosylation moieties should reflect mammalian glycosylation patterns. These results are intriguing in light of recent findings that a gp120 glycopeptide epitope bound to murine I-A and I-E elicited CD4^+^ T cell responses in immunized mice in a glycan-dependent manner (Sun et al., 2020).

**Figure S4.**
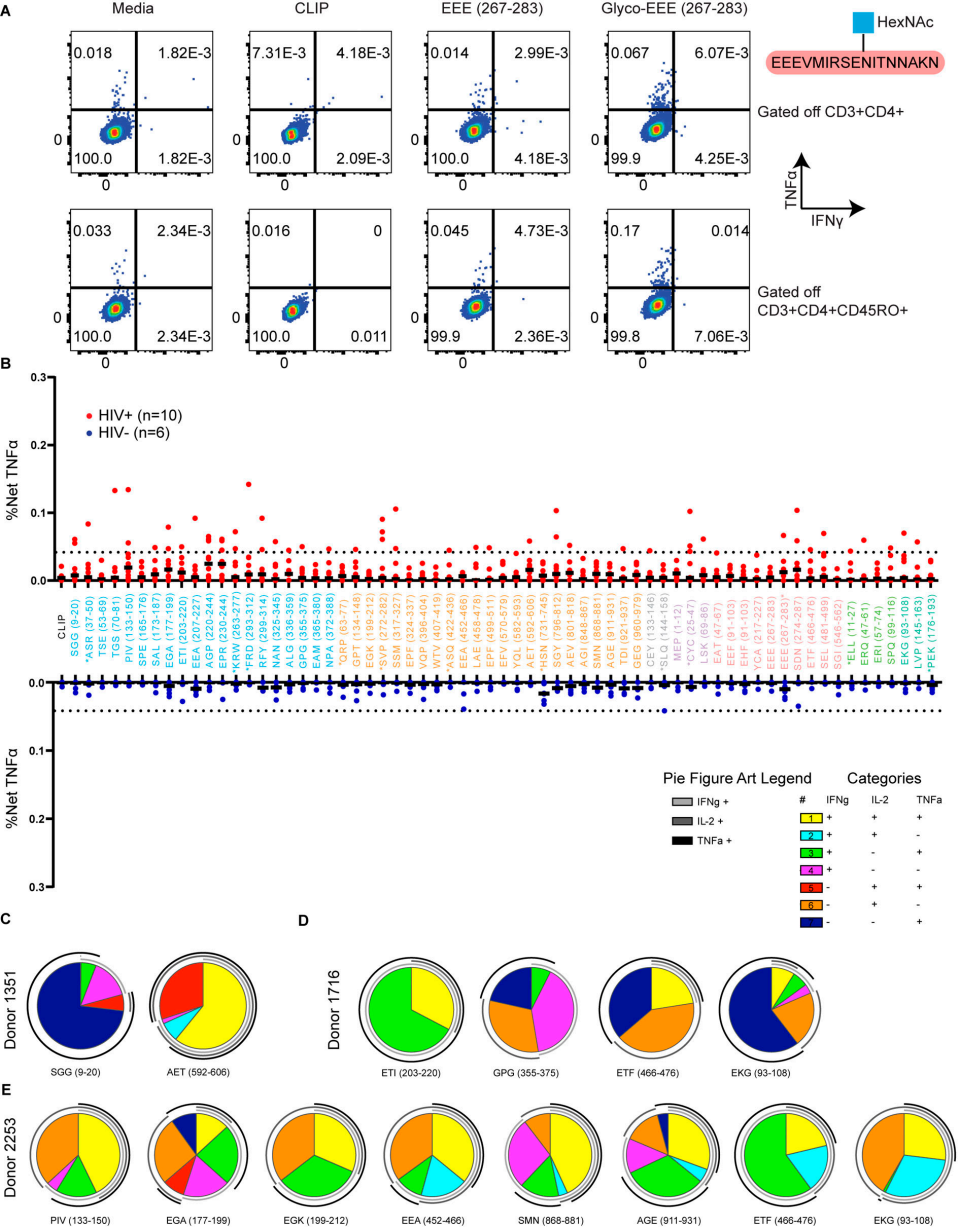
**DR1-restricted memory CD4**^**+**^ T cell response to a glycopeptide epitope, lack of responses to epitopes from DR1*01:01 HIV^**−**^ **donors, and polyfunctionality of responses. (A)** IFNγ^+^TNFα^+^ cytokine responses gated off of CD3^+^CD4^+^ (top) or CD3^+^CD4^+^CD45RO^+^ (bottom) cells are shown for Donor 3641 in response to media only, CLIP (irrelevant peptide), unglycosylated Env-EEE_267-283_, and glycosylated Env-EEE_267-283_. **(B)** Total TNFα induced across *n* = 6 HIV^−^ donors and *n* = 10 HIV^+^ donors from PBMC peptide-pulsing experiments, with specific activation percentages (net TNFα) corrected by subtracting the background TNFα from media stimulation to allow for comparison between both groups. A dotted line is drawn at the highest-magnitude net TNFα responses from HIV^−^ donors (0.042%). **(C–E)** CD4^+^ T cell cytokine secretion from DR1*01:01 PLWH was analyzed from Donors (C) 1351, (D) 1716, and (E) 2253 in response to peptides derived from cell-free processing (Table S4). Activation after stimulation in A–E was measured in single determinations due to the large number of cell-free-derived epitopes tested and the cell input required for testing polyfunctional cytokine responses by flow cytometry.

A glycan moiety located within an epitope’s core binding register may affect T cell recognition. In that sense, the DM-resistant glycoepitope EEE_267-283_ in BG505 is particularly interesting (Table S3). Glycopeptide EEE_267-283_ is modified with a single N-acetylglucosamine (Table S3). The modified Asn does not affect peptide/DR1 binding, as it was eluted from immunoprecipitated DR1 following cell-free processing. However, the glycan is close to or within the binding register for DR1, which is likely VMIRSENIT. This epitope is located on an external unstable loop making this more accessible to the MHC-II groove (Fig. S2 F). It also overlaps the binding footprints of >15 broadly neutralizing antibodies (bNAbs) that target the CD4 binding site (Fig. 7 B). Notably, one of 10 DR1+ PLWH tested (Donor 3641) showed a memory T cell cytokine response to the glycosylated but not unglycosylated form of EEE_267-283_ (Fig. S3 E and Fig. S4 A). Further studies will need to verify whether glycan-dependent T cell responses are observed in larger cohorts and if such responses impact humoral immunity. Aside from EEE_267-283_, we observed several examples of T/B cell epitope overlap at the CD4 binding site or gp120/gp41 interface (Fig. 7 B).

In addition to novel epitopes from Env, several epitopes identified by cell-free processing from the accessory proteins Vif, Tat, Rev, and Nef have not been reported, including the dominant, C-terminal Rev-SPQ_99-116_ epitope (Fig. 3 C) and Tat-MEP_1-12_ (Fig. 3 B). Overall, these results highlight the fact that by isolating antigen-dependent factors promoting epitope dominance with a minimalist cell-free processing system, one can identify a novel and potentially more informative set of T cell epitopes than those only obtained from overlapping peptide pulsing studies.

### HIV-1 epitopes from in vitro processing elicit memory CD4^+^ T cell responses in DR1^+^ PLWH

**Figure 8.**
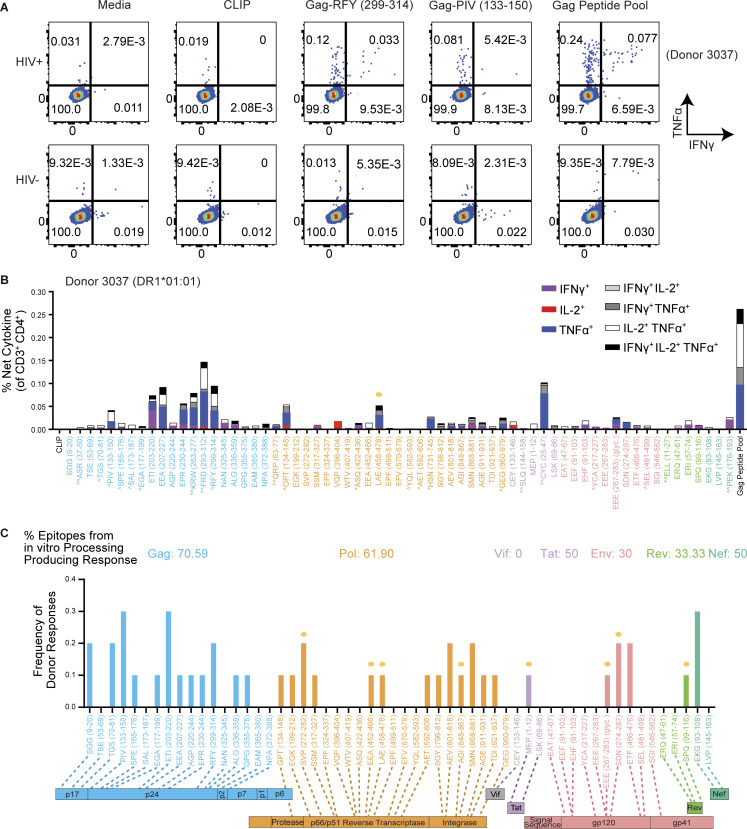
Epitopes from cell-free processing induce cytokine responses in DR1*01:01 HIV^**+**^ **individuals. (A)** Representative flow cytometry plots of IFNγ^+^TNF^+^ release from CD3^+^CD4 T cells after 16 h of PBMC incubation with Gag-RFY_299-314_ and Gag-PIV_133-150_ in a DR1*01:01^+^ HIV^+^ individual (Donor 3037) compared to a DR1*01:01^+^ HIV^−^ donor. Media, CLIP (irrelevant peptide), and Gag peptide pool controls are shown for comparison. **(B)** Representative single-, double-, and triple-positive (see legend) responses from the CD4^+^ T cells of Donor 3037 from ex vivo PBMC stimulation with 65 peptides across the HIV-1 proteome. Single-positive responses indicate cells that produced either IL-2, IFNγ, or TNFα, double-positive responses indicate cells that produced two of the three cytokines assessed, and triple-positive responses indicate cells producing all three cytokines. Asterisks denote peptides from the literature, while the remaining 56 peptides were identified from in vitro processing and selected for testing in this screen (Table S4). ^ indicates epitopes from the literature known to be restricted by HLA DR1*01:01. Gold circles indicate novel epitopes identified from cell-free processing that induced a response. Activation after stimulation was measured in single determinations. **(C)** Frequency of HIV^+^ donor responses to 56 of the epitopes obtained from cell-free processing as measured by IL-2, IFNγ, or TNFα positivity compared with CLIP within each donor. Responses were considered positive if they were polyfunctional and at least two of the cytokines measured represented a >2.95-fold increase in the magnitude of response relative to CLIP. The percent of epitopes from in vitro processing that produced a response is listed in the corresponding color. Responses to novel epitopes are indicated with a gold circle. Data in C were obtained from *n* = 10 HIV^+^ donors. Activation after stimulation was measured in single determinations due to the large number of cell-free-derived epitopes tested and the cell input required for testing polyfunctional cytokine responses by flow cytometry.

**Figure S5.**
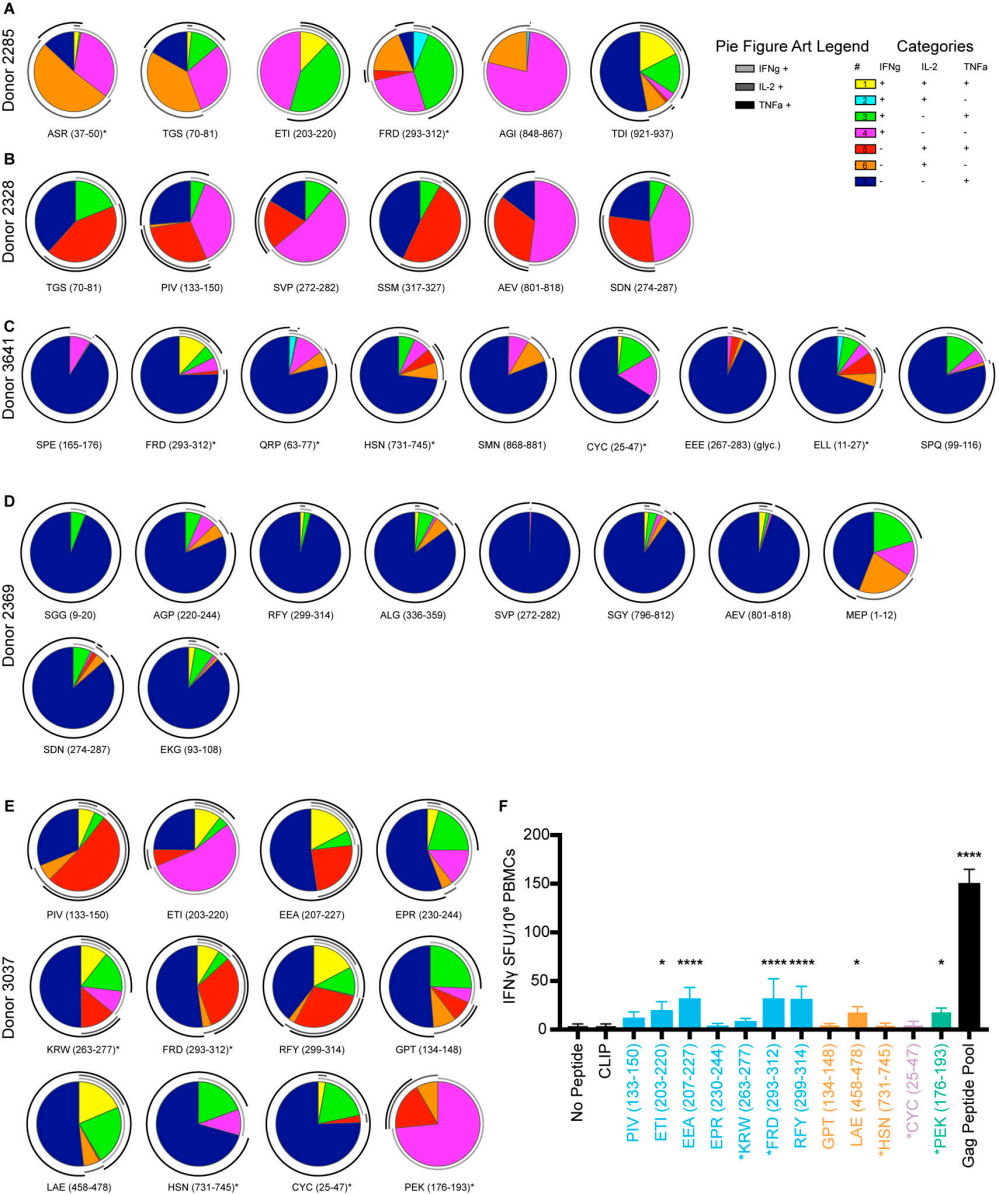
**Polyfunctionality of CD4**^**+**^ **T cell responses in PLWH to peptides derived from cell-free processing and IFNγ ELISPOT validation for Donor 3037. (A–E)** CD4^+^ T cell cytokine secretion from DR1*01:01 PLWH was analyzed from Donors (A) 2285, (B) 2328, (C) 3641, (D) 2369, and (E) 3037 in response to peptides derived from cell-free processing. Peptides are listed as in Table S4, with asterisks denoting control peptides identified in the literature. Double-positive cells were those that produced two cytokines. Double positive cells were calculated by the following strategies: (a) IL-2^+^TNFα^+^: Lymphocytes→ Single Cells→ Live^+^CD3^+^→ CD3^+^CD4^+^→ TNFα^+^ → IL-2^+^, and the percentage of IL-2^+^ cells was multiplied by the percentage of TNFα^+^ cells from the parent gate; (b) IFNγ^+^TNFα^+^: Lymphocytes→ Single Cells→ Live^+^ CD3^+^→ CD3^+^CD4^+^→ TNFα^+^→ IFNγ^+^, and the percentage of IFNγ^+^ cells were multiplied by the percentage of TNFα^+^ cells from the parent gate; (c) IL-2^+^IFNγ^+^ Lymphocytes→ Single Cells→ Live^+^CD3^+^→ CD3^+^CD4^+^→ IL-2^+^ IFNγ^+^ → TNFα^−^, and the percentage of TNFα^−^ cells were multiplied by the percentage of IL-2^+^ IFNγ^+^ cells from the parent gate. Single-positive populations were calculated by the following: (a) IL-2^+^: Lymphocytes→ Single Cells→ Live^+^CD3^+^→ CD3^+^ CD4^+^ → TNFα^−^ → IL-2^+^ IFNγ^−^, and the percentage of IL-2^+^ IFNγ^−^ cells were multiplied by the percentage of TNFα^−^ cells from the parent gate; (b) IFNγ^+^: Lymphocytes→ Single Cells→ Live^+^CD3^+^→ CD3^+^ CD4^+^ → TNFα^−^→ IL-2^−^ IFNγ^+^, and the percentage of IL-2^−^ IFNγ^+^ cells were multiplied by the percentage of TNFα^-^ cells from the parent gate; (c) TNFα^+^: Lymphocytes→ Single Cells→ Live^+^CD3^+^→ CD3^+^ CD4^+^ → TNFα^+^ → IL-2^−^IFNγ^−^, and the percentage of IL-2^−^ IFNγ^−^ cells were multiplied by the percentage of TNFα^+^ cells from the parent gate. Data gated in this manner was analyzed by SPICE and represented as pie charts in A–E. Responses depicted are to those peptides that induced CD4^+^ T cell cytokine responses that were 2.95-fold greater than the response to CLIP. Activation after stimulation in A–E was measured in single determinations. **(F)** IFNγ ELISPOT from Donor 3037 with data shown as spot forming units (SFU) per million PBMCs. Peptides utilized were those shown in E. Responses to negative controls (no peptide, CLIP) and positive control (Gag peptide pool) are shown as comparators, with six replicate wells run for each condition. Data represents two independent experiments. Significance difference relative to CLIP determined by one-way ANOVA with Dunnett’s test for multiple comparisons, *P < 0.05, ****P < 0.0001.

The above experiments detail the characteristics of peptides obtained from cell-free processing across the HIV-1 proteome for a single MHC-II allele. We evaluated whether these peptides (Table S4) could be presented in vivo by analyzing CD4^+^ T cell responses from DR1^+^ PLWH on suppressive combination antiretroviral therapy (cART; Table S5). Early administration of cART preserves T cell immunity (Altfeld et al., 2001; Le et al., 2013; Ndhlovu et al., 2019). Of 10 donors studied, five were treated during acute infection (<6 mo after infection; Table S5). CD4^+^ T cell responses were measured by intracellular cytokine staining for IFNγ, IL-2, and TNFα (Fig. S3 E and Fig. 8 A). As internal controls, we included a representative sample of previously published epitopes (Fig. 8 B, denoted in asterisks), as well as the irrelevant self-peptide CLIP_89-105_ (KMRMATPLLMQALPM) that binds to nascent DR1 (Fig. 8 A). Responses were confirmed with multiple cytokines and were not observed in cells from DR1^+^ HIV^−^ healthy donors (*n* = 6; Fig. S4 B). Responses were considered positive if they were polyfunctional (at least two or more cytokine responses; Fig. S4, C–E, and Fig. S5, A–E) and represented a greater than 2.95-fold increase in magnitude relative to the response to CLIP.

Of the donors tested, Donor 3037 displayed the greatest breadth of responses (Fig. 8, A and B). Donor 3037, who first tested positive for HIV-1 in 1979 (from a retrospective sampling of banked specimens), started on nucleoside reverse transcriptase inhibitor monotherapy in the late 1980s and on cART in 1998 (Table S5). Following development of drug-resistance mutations, a new and fully suppressive regimen was started in 2018. Despite the time interval between infection and suppressive therapy, Donor 3037 had polyfunctional responses to two of the three dominant DM-resistant epitopes identified in cell-free processing of p24: Gag-PIV_133-150_ and Gag-RFY_299-314_ (Fig. 8 B and Fig. S5 E), in addition to the parent epitope FRD_293-312_ described in the literature. FRD_293-312_, with the core epitope of Gag-RFY_299-314_, has been associated with viral control (Benati et al., 2016; Ranasinghe et al., 2012; Vingert et al., 2010). This donor also had a response to the overlapping epitopes Gag-ETI_203-220_/Gag-EEA_207-227_ (Fig. 2 B), which are accessible and unstable epitopes that represent the third most abundant p24 epitope from p24p2p7 processing (Fig. 4 A). Furthermore, donor 3037 showed a polyfunctional response to the novel RT epitope Pol-LAE_458-478_ (Fig. 2 E), which was the most abundant DM-resistant RT epitope from cell-free processing (Fig. 6 F). Responses to Gag-RFY_299-314_, Gag-FRD_293-312_, Gag-ETI_203-220_, Gag-EEA_207-227_, but not Gag-PIV_133-150_, were confirmed independently using an IFNγ ELISPOT (Fig. S5 F). Robust CD4^+^ T cell responses to these epitopes may have conferred some protective immunity and prevented progression to AIDS for 20 yr (Table S2).

**Figure 9.**
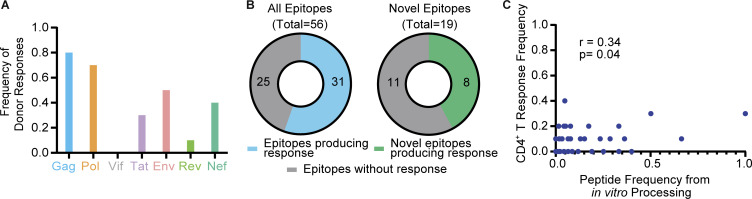
**Characteristics of CD4**^**+**^T cell responses from DR1*01:01^**+**^ **HIV**^**+**^ **individuals to epitopes identified from cell-free processing. (A)** Frequency of HIV^+^ donor responses to individual HIV-1 antigens are shown. **(B)** Number of epitopes producing a CD4^+^ T cell response of the 56 total and 19 novel epitopes tested in ex vivo stimulation experiments are shown. **(C)** Pearson correlation between peptide frequency from in vitro processing and donor CD4^+^ T cell response frequency across PLWH is shown. Peptide frequency was assessed by dividing PSMs for an epitope by the total MS spectra for a particular protein. r and P values are shown. Data in A–C were obtained from *n* = 10 HIV^+^ donors. Activation after stimulation was measured in single determinations.

Altogether, from 10 PLWH, we observed cytokine responses to a substantial fraction of epitopes identified by in vitro processing: Gag, 70.6%; Pol, 61.9%; Env, 30%; Vif, 0%; Tat, 50%; Rev, 33%; and Nef, 50% (Fig. 8 C). Responses were observed to 55.3% of all DM-resistant epitopes, including novel epitopes. Most responses were specific for Gag or Pol epitopes (Fig. 9 A). Of the 56 epitopes tested, 31 produced responses in cells from PLWH, including eight of the 19 novel epitopes tested here (Fig. 9 B). Some epitopes elicited very strong responses but in only a subset of donors. Thus, a minimalist cell-free system can uncover novel epitopes that are processed and presented in vivo.

If efficiency of epitope processing and presentation is a determinant of T cell responses, epitope abundance from cell-free processing may be reflected in the frequency of DR1-restricted responses to that epitope. The Gag-PIV_133-150_ epitope, which was one of the most frequently targeted by PLWH in our cohort (30%, Fig. 8 C), displayed the greatest abundance by peptide spectra (50.8 and 88% of DM-resistant epitopes from p24p2p7 and p24, respectively; Fig. 6, A and B), consistent with its structural characteristics (Fig. 5, A and B) and mutational profile (Fig. S3, A and B). Nef-EKG_93-108_, the most abundant DM-resistant Nef epitope by MS (Fig. 6 C), also induced CD4^+^ T cell responses in 30% of HIV^+^ individuals (Fig. 8 C). Overall, we observed a modest but significant correlation between peptide frequency from in vitro processing and CD4^+^ T cell response frequency (r = 0.34, P = 0.04; Fig. 9 C).

## Discussion

Our cell-free antigen processing system interrogates how structural features of an antigen and components of the MHC-II antigen processing pathway allow regions of an antigen to be efficiently processed and emerge as dominant. This contrasts with most epitope prediction algorithms that rely on peptide affinity for an MHC allele or T cell responses to overlapping peptides. Prediction algorithms based on peptide/MHC affinity or peptide-elution data (Abelin et al., 2017; Chen et al., 2017; Jurtz et al., 2017) fail to mimic environmental conditions that create epitope hierarchies, leading to variable success in predicting MHC-II epitopes. T cell responses to overlapping peptides used at non-physiologic micromolar concentrations may omit specific registers that are optimal for MHC-II binding (Godkin et al., 2001; Lovitch et al., 2006) and cannot account for posttranslationally modified epitopes. Additionally, studies relying on IFNγ ELISPOT assays overlook responses from individuals who have a limited CD4^+^ IFNγ response. Most importantly, as highlighted by Reinherz and colleagues, this “reverse immunology” approach only identifies previously recognized epitopes and may miss epitopes that could provide optimal control of infection (Keskin et al., 2015). This is especially relevant for HIV-1, where protective CD4^+^ T cell epitopes have been documented, but most individuals progress to chronic infection.

To overcome these limitations, we subjected nearly the entire HIV-1 proteome to our reductionist cell-free antigen processing system. We identified novel epitopes that could be targeted in future vaccine studies. Examples include several conserved DM-resistant Pol epitopes. CD4^+^ T cells specific for internal viral proteins can provide help to B cells in making antibodies to Env, as long as both are present in the viral particle internalized by the B cell (Milich et al., 1987; Russell and Liew, 1979). Targeting highly conserved intracellular epitopes from Gag and Pol proteins, rather than the more mutable Env, may prove beneficial for vaccine strategies seeking to induce robust neutralizing antibodies. One could envision a vaccine utilizing a viral vector containing Gag, followed by a heterotypic vector containing Pol, that engages T cell responses to both proteins and provides more breadth than Gag alone (Arunachalam et al., 2020; Liu et al., 2009). Indeed, recent preventative vaccine studies in the simian immunodeficiency virus model suggest a role for help from CD4^+^ T cells specific for intracellular viral proteins (Casimiro et al., 2005; Liu et al., 2009). Additionally, HIV^+^ individuals who developed neutralizing antibodies and spontaneously controlled HIV-1 viral loads to <2,000 copies/ml for >1 yr in the absence of cART had stronger CD4^+^ T cell responses to Gag than to gp120 (Ranasinghe et al., 2015).

In addition to novel Pol epitopes, we identified glycopeptide epitopes, highlighting the utility of unbiased, antigen-processing-based epitope discovery. While Env sequence variation certainly plays a role, the failure of overlapping peptide methods to detect glycopeptide responses may partly explain why fewer responses to Env have been documented compared to Gag or Nef (Kaufmann et al., 2004; Ranasinghe et al., 2012), despite the fact that Env can readily enter the exogenous and endogenous pathways of MHC-II processing following internalization (Byland et al., 2007; Callahan et al., 1993; LaBranche et al., 1995). We show here that HIV-1 Env epitopes that are glycosylated can bind to DR1, withstand DM-mediated displacement, and be presented to CD4^+^ T cells. Thus, some Env-derived epitopes may be glycopeptides not assayed by standard methods. Measuring responses to these epitopes may allow better correlations between antibody titers and Env-specific CD4^+^ T cell responses in vaccine studies (Pauthner et al., 2019; Sanders et al., 2015).

We made several observations regarding factors influencing immunodominance. We observed (a) epitope hot spots and similarities in epitopes from HIV polyproteins versus individual subunits; (b) the location of epitopes from HIV-1 proteins of known structure (p24 and accessory proteins) map to regions of low protein stability and high solvent accessibility; (c) DM influences epitope abundance; and (d) epitope selection patterns of specific HIV-1 proteins suggest that differences in processing/presentation efficiency influence T cell responses. Thus, structural features and binding to DR1 in the presence of DM allow prediction of epitope selection and opportunities for enhanced HIV-1 vaccine strategies.

### Cell-free processing highlights protein stability and epitope location in epitope dominance

Our cell-free processing system provides insights into antigen structure that predispose regions for capture by DR1 and subsequent processing and presentation. Most epitopes identified by cell-free processing—in particular for smaller proteins such as p24, Tat, Nef, and Vif—corresponded to regions of higher accessibility and lower protein stability. Such regions have an increased chance of unfolding, permitting capture by DR1. This relationship was less pronounced with larger, multi-domain proteins such as RT and Env, perhaps reflecting the increased inaccessibility of epitopes from these proteins in solution as well as computational limitations. Additionally, while certain regions may initially be less accessible and have higher stability, binding to DR1 in the MIIC compartment may facilitate protein unfolding, allowing previously “stable” regions to become unstable and available for processing (Sadegh-Nasseri and Kim, 2019). Likewise, in low pH and denaturing conditions, localized changes in protein folding and allosteric interactions during protein oligomerization may facilitate the exposure of previously stable regions, allowing for their capture and presentation by DR1. In the low pH and denaturing conditions utilized in our cell-free system, DR1 binding sites could be exposed regardless of original tertiary structure for larger proteins. Overall, our results show that more accessible and less regions of HIV-1 protein antigens tend to be more efficiently processed.

Several identified epitopes overlapped with B cell epitopes. T/B cell epitope overlap has been shown to either boost or suppress T cell responses to epitopes contained within an antibody footprint, as antibody binding can protect the epitope from degradation upon virion endocytosis or sterically block the processing of the epitope (Jaume et al., 2002; Simitsek et al., 1995; Watts and Lanzavecchia, 1993). These findings may provide insights into T cell immunodominance for current bNAb trials seeking to enhance virus-specific T cell responses (Niessl et al., 2020).

### Epitope abundance in the presence of DM provides clues for improved vaccine design

The presence of DM affected the relative abundance of certain epitopes, and we observed a significant correlation between relative peptide abundance by LC-MS/MS and frequency of CD4^+^ T cell responses in HIV^+^ individuals. Prior studies have shown that greater surface density of agonist pMHC-II both enhances T cell activation (Korb et al., 1999; Mirshahidi.et al., 2004; Mirshahidi et al., 2001) and reduces the duration of the T cell priming phase (Henrickson et al., 2008; Mempel et al., 2004). DM-resistant epitopes will be presented at higher cell-surface density and induce stronger T cell activation, irrespective of naive TCR repertoire and TCR affinities, simply due to their enhanced antigen processing efficiency. It is interesting to also consider antigen density in the context of T follicular helper cell (Tfh) interactions with B cells. Tfh regulates the number of cell cycles that B cells undergo in the dark zone of the germinal center reaction, in a manner proportional to the number of cognate pMHC-II presented by those B cells (Gitlin et al., 2014). Thus, abundantly presented HIV-1 epitopes may drive Tfh responses that promote enhanced somatic hypermutation and the induction of neutralizing antibodies or even bNAbs in HIV^+^ individuals. Examining CD4^+^ T cell responses to abundant versus less abundantly presented epitopes may provide insights into optimal sequential vaccination regimens that induce bNAbs.

Some candidate HIV-1 vaccines use viral vectors to deliver multiple HIV-1 antigens in addition to Env (Liu et al., 2009). However, our results show protein stability and epitope competition can influence epitope selection. For example, the highly conserved Gag-RFY_299-314_ epitope, associated with viral control (Ranasinghe et al., 2012), was not observed from the Gag polyprotein processing but was observed at low abundance from processing of the p24 subunit alone. Low PSMs of Gag-RFY_299-314_ may reflect its location within a stable region of p24, which may undergo a delay in unfolding and be kinetically outcompeted by epitopes from less stable regions. Elite controllers may have developed compensatory mechanisms to sense lower levels of protective epitopes, including enrichment for public TCRs that have a high affinity for the extended Gag-293 epitope (Benati et al., 2016; Vingert et al., 2010). However, inducing responses to protective epitopes in non-controller individuals may be affected by epitope processing efficiency. If Gag-RFY_299-314_ epitope is inefficiently processed, as our results suggest, then including the whole Gag polyprotein may actually reduce the presentation of this conserved epitope. In vaccine strategies geared toward inducing CD4^+^ T cell responses to highly conserved epitopes, competition from other epitopes within the same protein and inefficiencies in antigen processing should be considered.

While most identified epitopes induced responses by CD4^+^ T cells from PLWH, some did not. This may indicate the following: (1) the epitope was not presented in vivo, potentially due to competition from other HIV-1 proteins; (2) the epitope was presented and primed CD4^+^ T cells that became exhausted or anergic; (3) the individual’s naive precursor and/or memory CD4^+^ T cell frequency for the pMHC-II was limited (Campion et al., 2014; Moon et al., 2007); (4) the protein from which the epitope is derived is expressed at a relatively lower level, leading to less peptide presentation; (5) the viral variant that primed these CD4^+^ T cells has sequence differences in the epitope; or (6) our cohort size may not have captured the full spectrum of responses to all the HIV-1 epitopes tested here. Future studies with larger cohort sizes may improve the chance of detecting positive responses to such epitopes. Larger cohorts may also provide insights into whether antigen-dependent factors predict epitopes that may be dually recognized by CD4^+^ T cells and very rare MHC-II restricted CD8^+^ T cells that have been observed in humans (Ranasinghe et al., 2016). Finally, studies with larger cohorts of PLWH would help establish whether the correlation we observed between epitope abundance and CD4^+^ T cell response frequencies is enhanced, which would have significant implications for understanding mechanisms of immunodominance and improved vaccine development.

While this study provides several insights into factors influencing epitope selection, there are a few limitations. A potential limitation of this approach is the preferential activation and HIV infection of HIV-specific CD4^+^ T cells (Douek et al., 2002). Recent studies have shown that some clones of HIV-infected, HIV-specific CD4^+^ T cells persist and proliferate in PLWH on cART (Collora et al., 2022; Simonetti et al., 2021). Nevertheless, it is important to note that in the setting of preventative vaccines, the use of novel epitopes identified with this system may provide better T cell help for a neutralizing antibody response that would prevent any infection of CD4^+^ T cells. In the context of a therapeutic vaccine, the use of epitopes identified with this system may allow the generation of a broader CD4^+^ T cell response including epitopes that were not targeted by the initial response to infection in PLWH. If the immunization is done in the setting of cART, no new cells will be infected.

Our use of ex vivo peptide stimulation experiments to validate the epitopes obtained from cell-free processing, while commonly used to assess for CD4^+^ T cell memory responses to pathogen-derived peptides, does not incontrovertibly demonstrate in vivo processing and presentation of these antigens. Tracking of tetramer-positive CD4^+^ T cells after the onset of acute infection and functionally characterizing isolated tetramer-positive cells for proliferation and cytokine release in response to peptide or whole protein antigens would provide additional evidence to support in vivo presentation during natural infection. Additionally, since the cell-free system obviates the need for identifying the nature of the APC, it is unclear whether infected CD4^+^ T cells present these peptides directly or whether DCs indirectly present these epitopes during the course of natural infection (Addison et al., 2022). Finally, the cell-free processing system in this study generates an HIV-1 peptidome for a single MHC-II allele, DR1*01:01, and as such is not completely generalizable. However, results from these analyses can provide insights into overall epitope selection trends across other HLA alleles.

### Conclusions and future studies

Altogether, these results show that epitope selection based on resistance to DM-mediated dissociation and the structure of the antigen influence epitope selection leading to memory CD4^+^ T cell responses in PLWH. The relative abundance of epitopes presented by DR1 in the presence of DM was reflected by the frequencies of observed CD4^+^ T cell responses. Future studies investigating the relative contribution of each APC to epitope-specific CD4^+^ T cell responses may be achievable in humanized mouse studies. Additionally, future studies that incorporate multiple HIV-1 proteins in the cell-free assay could reveal which epitopes dominate when the input antigen is a whole virion (Kim et al., 2017). In vitro processing of proteins produced from mosaic vaccines or viral-vectored vaccines could also provide information on the presentation of specific HIV-1 epitopes associated with protection. Given the central role of CD4^+^ T cells in coordinating adaptive immunity, understanding epitope selection of vaccine antigens is invaluable not only for HIV-1 but also for other challenging pathogens such as malaria and *Mycobacterium tuberculosis*, and for understanding differential vaccine responsiveness to SARS-CoV-2.

## Materials and methods

### Experimental model and subject details

#### Human subjects

Functional assays were performed using peripheral blood mononuclear cells (PBMCs) from 10 DR1^+^ (HLA-DR1*01:01) HIV^+^ individuals from the University of California, San Francisco (UCSF) Study on the Consequences of the Protease-Inhibitor Era (SCOPE) cohort (see Table S5 for additional details on study participants). All study subjects provided written informed consent before participation in the study, and the study was approved by the UCSF Institutional Review Board. Deidentified PBMCs from six DR1^+^ (HLA-DR1*01:01) HIV^−^ individuals were obtained via leukapheresis samples (STEMCELL) as controls.

#### Cell lines

Sf9 and Hi5 cells were used to produce recombinant DR1 and DM. Cells were grown as previously described (Kim et al., 2014; Narayan et al., 2007) in ISFM media (Cleveland et al., 2014; Inlow et al., 1989) at a density of 0.3–0.5 million cells/ml at 27°C, with regular passaging every 3–4 d.

### Method details

#### Production of recombinant proteins

Soluble HLA-DR1*01:01 and DM were produced as described (Kim et al., 2014; Narayan et al., 2007). Baculovirus DNA (BaculoGold; PharMingen) and transfer vectors carrying DR α- and β-chains were transfected together into Sf9 insect cells to produce recombinant viruses. Recombinant viruses were passaged three times before being used to infect High Five cells in ISFM media (Cleveland et al., 2014; Inlow et al., 1989). DR1 proteins were purified from culture supernatants using immunoaffinity chromatography with a monoclonal antibody L243 to DR1 (purified from HB-55 hybridoma; American Type Culture Collection). Soluble DM was also expressed by High Five cells transduced with baculovirus containing the extracellular domains of genes encoding the α- and β-chains of human DM. The truncated DM α- and β-chains were modified to contain the FLAG epitope (DYKDDDDK) and c-Myc epitope (EQKLISEEDL), respectively, at their C-termini. DM protein was purified from culture supernatants with a monoclonal antibody to M2 (anti-FLAG) agarose resin (Sigma-Aldrich) and eluted with 5 mg/ml FLAG peptide (Sigma-Aldrich) in tris-buffered saline. DM was further concentrated and buffer exchanged into citric phosphate buffer, pH 6, with 05% wt/vol sodium azide and stored in aliquots at −80°C.

#### Cell-free processing assay

Cell-free processing experiments were conducted as described (Hartman et al., 2010; Kim et al., 2014). Specifically, on day 0, 20 μM DR1 was preincubated with 400 μM HA(Y308A), which forms short-lived complexes with DR1 and generates a peptide-receptive DR1 conformation (Kim et al., 2014). After incubation overnight at 37°C, 325 pmol of peptide-receptive DR1:Y308A was added to 750 pmol protein antigen in the presence or absence of 162.5 pmol of DM, together in citrate-phosphate buffer (pH 5–5.2) with 6 mM L-Cysteine. This mixture was incubated at 37°C for 3 h. After this time, a cathepsin digestion mixture consisting of 90 pmols Cathepsin B (bovine spleen, Sigma-Aldrich), 90 pmols cathepsin H (human liver, Calbiochem), and 38 pmols cathepsin S (human recombinant protein produced in *Escherichia coli*, Calbiochem), as well as 4 mM EDTA were added to the reaction for an additional 2 h. At the end of cathepsin digestion, the pH of the assay was adjusted to 7.4 with equal volumes of 2 M sodium dibasic buffer and 1X PBS, and 10 μM iodoacetamide was added to inhibit cathepsin activity. Peptide-bound DR1 molecules were immunoprecipitated with HLA-DR–specific mAb (L243)-conjugated Sepharose beads for 1 h at 4°C. DR1-bound beads were washed with PBS and water, and pMHC-II molecules were eluted from the antibody-conjugated beads with mild acid elution (0.1% TFA). Peptides were subsequently eluted from DR1 with 1% TFA and 40% MeOH/1% TFA and physically separated from MHC molecules using a 10 kD MWCO filter (Millipore) before being lyophilized dry.

#### Hydrophilic interaction LC (HILIC) cleanup

Peptides were cleaned for MS by HILIC using columns (HILIC TopTips) containing poly (2-sulfoethylaspartamide)-silica membranes (Alpert and Andrews, 1988). Lyophilized peptides were resuspended in 85% acetonitrile (ACN) in 15 mM ammonium formate (NH_4_HCO_2_) at room temperature. HILIC TopTips were conditioned by washing with 0, 2, 10, and 85% ACN/NH_4_HCO_2_. Sample was added to the column in 10 μl increments. Columns were washed with 85% ACN/NH_4_HCO_2_, and bound peptides were eluted first in 10%, then 2%, and finally 0% ACN/NH_4_HCO_2_. Peptides were lyophilized and identified by LC-MS/MS.

#### MS

Peptides were separated via reverse-phase chromatography with an Easy nLC 1000 (Thermo Fisher Scientific), using a gradient of 2–90% ACN/formic acid over 60 min at 300 nl per min on a C18 column packed with MAGIC AQ C18 at 3µm, 100 Å (MICHROM Bioresources, Inc.). Eluting peptides were sprayed onto the nano-LC-Q-Exactive Plus Orbitrap (Thermo Fisher Scientific) through a 10-µm integrated emitter tip at 2.2 kV. Survey scans (full mass spectra) were acquired on the Orbitrap within 350–1,800 D m/z using the data-dependent Top 10 method with dynamic exclusion of 10 s. Precursor ions were individually isolated with 1.6 D and fragmented (MS/MS) using high energy collisional dissociation activation collision energy 28. Precursor and fragment ions were analyzed at a resolution of 140,000/35,000 at 200 D. Automatic gain control target 3×e6 max IT 60 ms and automatic gain control target 1×e5, mx IT250 ms for parent and fragment ions, respectively.

#### MS data analysis

Tandem MS2 mass spectra were analyzed by Proteome Discoverer (v1.4 Thermo Fisher Scientific) in three ways, using 3Nodes: common, Xtract (spectra are extracted, charge state deconvoluted, and deisotoped using Xtract option, at resolution 105 K at 200 D) and MS2 Processor. MS/MS spectra from 3Nodes were analyzed with Mascot v.2.5.1 Matrix Science (www.matrixscience.com) using a Custom Database (2015RefSeq_72r_human with added client proteins database) consisting of sequences from all HIV-1 proteins tested (Table S6) as well as molecules of the in vitro processing system (MHC-II, DM, and cathepsins), and a concatenated decoy database, specifying the following search parameters: “no enzyme,” precursor mass tolerance of 8 ppm, fragment mass tolerance of 0.02 D, and variable modifications (cysteine carbamidomethylation, methionine oxidation, and other custom modifications). “No enzyme” was used to detect peptides generated by cleavage after any residue. Mascot “.dat” files were compiled in Scaffold. Scaffold Viewer Software (version Scaffold_4.8.9, Proteome Software) was used to validate MS/MS-based peptide and protein identifications. Scaffold uses the Protein Prophet algorithm to assign probabilities for protein identification (Nesvizhskii et al., 2003) and the PeptideProphet or LDFR algorithm to assign probabilities for peptide identification. Peptide identifications used for analysis were those that could be established at 95% probability to achieve a false discovery rate (FDR) <1% by the Peptide Prophet algorithm (Keller et al., 2002) with Scaffold delta-mass correction. Proteins used for analysis were those that could be identified at 99% probability to achieve an FDR of <1% and contained more than one identified peptide (Welsh et al., 2020). Spectral counting via Scaffold was done using the Total Spectra method, which uses the sum of all spectra associated with a specific protein within a sample, and also includes spectra shared with other proteins. Peptides identified from PSMs after in vitro processing were grouped into clusters based on shared start and end residues and their extent of overlap. DR1 contains a nine-residue binding groove, between the P1 and P9 anchor positions, so we considered the overlap of residues within this region. If two peptides had different anchor residues (i.e., Tyr, Phe, Pro, Ile, Leu) or lacked a nine-residue overlap within the DR1 binding groove, they were considered distinct epitopes. For each epitope cluster, the edges of each candidate epitope were defined using the peptide with the greatest number of PSMs, which is referred to as the core epitope in the relevant figures. Epitopes that were considered DM sensitive were those which were detected in the +DR1 only condition by the above criteria but could not be detected at an appropriate significance in the +DR1/+DM condition (see Fig. S1 B for an example).

#### Glycopeptide identification

Glycopeptides were identified using the GPQuest software (Toghi Eshghi et al., 2015). Briefly, LC-MS/MS raw files were converted to mzML files using the msconvert tool in ProteoWizard with peak picking function. In GPQuest search, mass tolerance of MS1 and MS2 levels were 10 and 20 ppm, respectively. In-house N-linked glycan database with 277 compositions and O-linked glycan database with 83 compositions from Functional Glycomics Gateway (CFG, http://www.functionalglycomics.org/fg/) were used. The peptide database included 2,042 human N-linked glycopeptides and tryptic peptides of gp120 with two missed cleavages. MS/MS spectra were filtered to have at least three oxonium ions, and an oxonium ion at 204 m*/z* was mandatory. Information on oxonium ions was used to predict the type of glycosylation that facilitated data interpretation. A modified Morpheus score was calculated using singly charged −b and −y peptide fragment ions and peptide + glycan fragment ions, and epitopes with a score >6.16 was used to identify hits. FDR was calculated as described previously. The identified glycopeptides were manually checked to ensure the quality of MS/MS spectra for identification.

#### Accessibility and structural stability analysis

Solvent ASA were obtained by inputting PDB structures in Table S1 into the PDB PISA tool. The ASA of each residue for the relevant protein chain in an interface could be extracted. The stability constants for each residue were also obtained from the respective PDB structures for each protein using the COREX/BEST web program (Hilser and Freire, 1996; Vertrees et al., 2005). Specifically, the algorithm models equilibrium conformational fluctuations of a protein to generate an ensemble of microstates, capturing the partial unfolding observed from hydrogen exchange and NMR relaxation experiments (Bai et al., 1995; Hilser and Freire, 1997). The COREX algorithm apportions the protein into “folding windows” that are overlaid onto the high-resolution crystal structure to generate a collection of differently folded states of the protein. The thermodynamic contribution of each Boltzmann-weighted state in this ensemble to the overall thermodynamic properties of the protein can be used to obtain a probability of local unfolding for different regions of the protein structure.

To calculate the free energy of each microstate, the calorimetrically parameterized enthalpy and heat capacity of the state are obtained from the change in solvent-accessible area from protein unfolding from the crystal structure (Murphy and Freire, 1992). The total entropy of the state is determined by the sum of the calorimetrically parameterized solvation entropy (due to the change in solvent accessibility) and the weighted conformational entropy (estimated from molecular dynamics simulation; D’Aquino et al., 1996; Lee et al., 1994). These thermodynamic properties can be used to determine the free energy of the microstate and thus, the statistical weight of that state in contributing to the overall ensemble. From this, the algorithm determines stability constants, essentially an equilibrium constant for folding, which provide the summed probability of states in which a residue is in a folded conformation over the number of states in which residues are in an unfolded conformation. These stability constants are the primary data plotted in Fig. 5. Residues that are more stable will be folded in most high-probability states while residues with low stability constants will be unfolded in most high-probability states. This method of calculating protein stability based on high-resolution structural data has been validated experimentally by hydrogen/deuterium exchange (Hilser and Freire, 1996; Hilser et al., 1998, 2006; Pan et al., 2000; Whitten et al., 2005) as well as NMR-monitored acid denaturation and cold denaturation of proteins (Babu et al., 2004; Hilser and Freire, 1996; Liu et al., 2012; Whitten et al., 2006).

For our analyses, a window size of eight residues, minimum window size of four, and 10,000 microstates per partition were selected for generating the ensemble for each protein. Exceptions were for the PDB structures 4ZMJ (BG505 SOSIP) and 1HMV (unliganded p66 and p51 subunits, analyzed separately), where a window size of 10 and 9 were used, respectively, to accommodate the larger size of the proteins. These represent an increased degree of sampling as compared to the defaults for the Monte Carlo option provided by the COREX/BEST server. All other unspecified parameters were default values. Thus, each protein ensemble was composed of ∼8 × 10,000 = 80,000 partially unfolded states.

#### Sliding scale analyses

A sliding scale of random epitopes was generated across the length of the respective protein to quantify whether an epitope’s solvent accessibility or epitope stability differed significantly from random epitopes throughout the protein (see Table S8 for an example). Each epitope of interest contains accessibility and stability values associated with every residue. These values can be averaged to give an accessibility or stability score for the epitope. To compare this score to randomly generated epitopes across the protein, we customized a sliding scale analysis for each epitope. If the epitope was a 15-mer, then a sliding scale of random 15-mers spanning the protein—excluding any 15-mers that touched the epitope of interest—was generated. Since each random 15-mer has accessibility and stability constants associated with each residue, it can also be assigned an accessibility and stability score by taking the average of these values.

The accessibility and stability scores for the entire set of random 15-mers can then be averaged and compared to the accessibility/stability score for the 15-mer epitope of interest. Thus, the random set was an internal control for each epitope, specific for the epitope’s complete protein. If the distribution of scores from the random 15-mers was normal, then a one-sample *t* test was used to quantify statistical significance between the mean of the distribution versus the value of the specific epitope. If the distribution of average accessibilities or stabilities was non-normal, then the Wilcoxon Signed Rank tests was used to quantify statistical significance between the median of the distribution compared to the value of the specific epitope. A P value of <0.05 was considered significant. Overall, this strategy provided a quantification for understanding how extreme a particular accessibility or stability score of an epitope was from the sample mean or median. This same analysis was conducted for target epitopes of different lengths (17-mers, 18-mers) for each of the HIV proteins. Distributions of the random sliding scale epitopes that excluded the epitope of interest were visualized to ensure that the overall distribution was unaltered before performing statistical analyses.

#### Mismatch analysis

To determine the level of conservation of residues within an epitope, the HXB2 protein sequence was compared to a collection of sequences from patients in the Los Alamos National Laboratory (LANL) HIV sequence database to assign a number of mismatches counted to each residue within a protein. The protein sequences downloaded from the database were of HIV subtype B, from plasma, and only included sequences that were 0% non-ACGT. Only one sequence per patient was included.

In addition to mismatches at the single residue level, we assessed the prevalence of full epitopes by aligning full epitope sequences to collections of patient sequences from LANL as described. Epitopes were aligned using an adapted Boyer-Moore alignment algorithm. Epitope prevalence was assessed in the context of zero permitted mismatches or one permitted mismatch between the epitope sequence and the collection of patient sequences. Data are shown as the percentage of patient sequences which contain the epitope.

#### Peptide synthesis

All peptides (Elim Biopharmaceuticals and JPT) were reconstituted at 5 mM in a solution of 50% dimethylformamide (Sigma-Aldrich) and 50% diethyl pyrocarbonate–treated water.

#### PBMC isolation

HLA-typed PBMCs from DR1^+^ HIV^−^ healthy donors (Stem Cell) and DR1^+^ HIV^+^ participants from the UCSF SCOPE cohort were isolated using a Ficoll gradient and cryopreserved. Cryopreserved PBMCs were thawed and rested for 2–5 h at 37°C in R10 medium (RPMI 1640 with 10% FBS and 1% penicillin/streptomycin) before use in stimulation assays.

#### Ex vivo stimulations

Rested cells were washed and plated into a 96-well U-bottom plate at a range of 5-1 × 10^6^ cells per well, depending on cell recovery. Cells were stimulated with 5 μM of the peptides listed in Table S4 for 16–18 h in the presence of 10 μM T20 (National Institutes of Health AIDS Reagent Program) and 1 μg/ml brefeldin A (BD Biosciences) at 37°C and 5% CO_2_. Peptides selected for the screen were generally DM-resistant epitopes that were dominant from LC-MS/MS, as well as a single control peptide per HIV-1 protein from the literature that had been documented to induce CD4^+^ T cell responses in the 2018 LANL Database. Cells treated with phytohemagglutinin (0.5 μg/ml; Remel Inc.), HIV-1 Consensus B Gag peptide pool (1 μg ml^−1^ per peptide; JPT), or HIV-1 Consensus Subtype B Nef peptide pool (1 μg ml^−1^ per peptide; JPT), served as positive controls, while cells stimulated with human short CLIP_89-105_ peptide (Elim Biopharm) or unstimulated (media only) served as negative controls. After washing 1× with PBS, PBMCs were incubated in Fc block (BD Biosciences) for 15 min at 23°C to prevent non-specific binding. Cells were stained with a viability dye and surface markers (15 min, 4°C) followed by intracellular cytokine staining (ICS). ICS was performed using the fixation/permeabilization solution kit (BD Biosciences) according to the manufacturer’s protocol, and BV Brilliant Stain Buffer (BD) was used during ICS at 10 μl/test. Samples were acquired on an LSRII flow cytometer (BD; see Fig. S3 for antibody staining panel). Negative controls were used together with fluorescence minus one controls to set gates for analysis with FlowJo software (Treestar).

#### ELISPOT

IFNγ ELISPOTs were performed using the ELISpot Pro: Human IFN-γ kit (3420-2HST; Mabtech) according to the manufacturer’s protocol. 250,000 PBMCs from SCOPE participant 3037 were plated per well in RPMI with 10% FBS and antiretrovirals (tenofovir and emtricitabine). The PBMCs were cultured for 20 h with 5uM peptide (Elim Biopharm) or 1 μg/ml/peptide HIV-1 Consensus B Gag peptide pool (JPT Peptide Technologies, PM-HIV-CONB). The plates were read by a blinded independent investigator using the AID iSpot Spectrum Reader. Six replicate wells were run for each condition, and data are representative of two independent experiments. pi Significant difference relative to CLIP determined by one-way ANOVA with Dunnett’s test for multiple comparisons, *P < 0.05, ****P < 0.0001.

#### Quantification and statistical analysis

For all antigens tested in the cell-free system, at least two independent experiments were performed, one with both DM and DR and one with only DR. For ex vivo stimulation experiments, PBMCs were assayed from *n* = 10 HIV^+^ on suppressive cART and *n* = 6 HIV^−^ DR1*01:01-expressing donors. Because of the limited nature of samples (PBMCs from HIV^+^ individuals), the large number (56) of cell-free derived epitopes being tested, and the cell input required for testing polyfunctional cytokine responses by flow cytometry, activation after stimulation was measured in single determinations. Simplified Presentation of Incredibly Complex Evaluation software was used to analyze flow cytometry data on T cell polyfunctionality as previously described (Roederer et al., 2011). The ELISPOT analysis represents data from two independent experiments. For box-and-whiskers plots depicting accessibility and stability metrics, normally distributed data were subject to a one-sample, two-tailed t-test, and non-normally distributed data were subject to a two-tailed Wilcoxon Signed Rank Test, comparing the mean (*t* test) or median (Wilcoxon Signed Rank) of the random epitope distribution to the mean ASA or stability of the epitope of interest. Statistical details of experiments can be found in the individual figure legends. Significance of all P values reported are as follows: *P < 0.05; **P < 0.01; ***P < 0.001; ****P < or = 0.0001. NS, not significant. Statistical analyses were performed in GraphPad Prism 8.0 or Matlab.

### Online supplemental material

Fig. S1 provides examples of the extracted base peak chromatographs from LC-MS/MS following cell-free processing and depicts how relative PSMs identified from LC-MS/MS can be displayed via a heat map. Fig. S2 provides accessibility and stability trends for HIV-1 proteins. Fig. S3 depicts the mutational characteristics and limit of detection for cell-free epitopes, as well as a representative gating strategy for assessing CD4^+ ^T cell responses from PLWH. Fig. S4 illustrates a glycopeptide-specific memory CD4^+^ T cell response and the lack of responses observed in healthy donors, as well as polyfunctional CD4^+^ T cell responses detected in three out of 10 HIV^+^ donors. Fig. S5 depicts polyfunctional CD4^+^ T cell responses in the remaining HIV^+^ donors as well as IFNγ ELISPOT results from Donor 3037. Table S1 describes the HIV-1 proteins subjected to cell-free processing. Table S2 lists epitopes obtained from cell-free processing that were reported in previous studies. Table S3 lists glycopeptides obtained from cell-free processing identified by GPQuest. Table S4 lists select peptides identified from cell-free processing. Table S5 describes the clinical characteristics of PLWH in the study. Table S6 lists sequences of proteins utilized in the cell-free processing system. Table S7 provides raw data obtained from cell-free processing (Excel). Table S8 provides an example of COREX analysis for Myr-MA (Excel). Table S9 provides the CD4^+^ T cell responses from PLWH (shown as fold change over CLIP) in ex vivo stimulations (Excel).

## Supplementary Material

Table S1 shows HIV proteins subjected to cell-free processing.

Table S2 shows epitopes obtained from cell-free processing reported in previous studies.

Table S3 shows glycopeptides obtained from cell-free processing identified by GPQuest.

Table S4 shows select peptides identified from cell-free processing.

Table S5 shows clinical characteristics of PLWH in study.

Table S6 shows sequences of proteins utilized in the cell-free processing system.

Table S7 provides raw data obtained from cell-free processing (Excel).

Table S8 provides an example of COREX analysis for Myr-MA (Excel).

Table S9 provides the CD4^+^ T cell responses from PLWH (shown as fold change over CLIP) in ex vivo stimulations (Excel).

## Data Availability

All data supporting the findings of this study are available within the paper or the supplementary materials and from the lead contact upon request. All original code used for epitope analysis has been deposited at Zenodo and is publicly available at https://doi.org/10.5281/zenodo.6360804. Further information and requests for reagents generated or used in this study are available upon request from the lead contact, Scheherazade Sadegh-Nasseri (ssadegh@jhmi.edu).
