# Supplementary material for: A cell-free antigen processing system informs HIV-1 epitope selection and vaccine design
Source: J Exp Med. 2023 Apr 14;220(7):e20221654. doi: 10.1084/jem.20221654 (PMC10114365; doi:10.1084/jem.20221654)
Supplement: Table S1 — shows HIV proteins subjected to cell-free processing. [file JEM_20221654_TableS1.docx]

**Table S1: HIV Proteins Subjected to Cell-Free Processing**

| **Protein** | **Subunit Name** | **Protein Antigen Strain** | **PDB Structures for PDB-PISA & COREX Analysis** |
| --- | --- | --- | --- |
| Myristoylated Matrix (MyrMA) | p17 | NL4.3 | 1UPH |
| Capsid-Spacer 1- Nucleocapsid (CA-SP1-NC) | p24-p2-p7 | NL4.3 | 1E6J; 4XFX |
| Capsid (CA) | p24 | HXB2 | 1E6J; 4XFX |
| Protease (PR) | ---- | HXB2 | ----- |
| Integrase (INT) | ---- | NL4.3 | 1EX4 |
| Reverse transcriptase (RT) | ---- | Pt. isolate | 1HMV |
| Envelope (Env) gp120 | gp120 | JR-FL | 4ZMJ |
| Envelope trimer | gp140 or BG505 SOSIP | BG505 | 4ZMJ |
| Nef | ---- | HXB2 | 1AVZ |
| Rev | ---- | HXB2 | 2X7L |
| Tat | ---- | IIIB | 3MIA |
| Vif | ---- | HXB2 | 4N9F |
