# Supplementary material for: A cell-free antigen processing system informs HIV-1 epitope selection and vaccine design
Source: J Exp Med. 2023 Apr 14;220(7):e20221654. doi: 10.1084/jem.20221654 (PMC10114365; doi:10.1084/jem.20221654)
Supplement: Table S2 — shows epitopes obtained from cell-free processing reported in previous studies. [file JEM_20221654_TableS2.docx]

**Table S2: Epitopes Obtained from Cell-Free Processing Reported in Previous Studies**

| **Protein** | **Epitope Cluster From Cell-free Processing** | **Epitope From Literature*** | **PubMed ID** |
| --- | --- | --- | --- |
| MyrMA | SGG_9-20_ (SGGELDKWEKIR) | SGGELDRWEKIRLRPGGK | 23793098, 21047960 |
| MyrMA | TSE_53-69_ (TSEGCRQILGQLQPSLQ) | EGCRQILGQLQPSLQTGS | 21047960 |
| MyrMA | TGS_70-81_ (TGSEELRSLYNT) | TGSEELRSLYNTVATLY | 23793098, 19165342 |
| p24 | QNY/PIV_130-151_ (QNYPIVQNLQGQMVHQAISPRT) | PIVQNIQGQMVHQAISPRTLNA | 9367954 |
| p24 | SPE_165-176_ (SPEVIPMFSALS) | AFSPEVIPMFSALSEGA | 22031937, 15078927 |
| p24 | EGA_177-199_ (EGATPQDLNTMLNTVGGHQAAMQ) | MFSALSEGATPQDLNTMLNT  PQDLNTMLNTVGGHQ | 17169395 |
| p24 | ETI_203-220_ (ETINEEAAEWDRLHPVHA) | LKETINEEAAEWDRVHPA  ETINEEAAEWDRVHPVHA | 17169395, 12517980 |
| p24 | EEA_207-227_ (EEAAEWDRLHPVHAGPIAPGQ) | AAEWDRLHPVHAGPIA | 23793098, 21047960 |
| p24 | AGP_220-244_ (AGPIAPGQMREPRGSDIAGTTSTLQ) | IAPGQMREPRGSDIA | 21047960 |
| p24 | NNP_252-259_ (NNPPIPVG) | TNNPPIPVGEIYKRWIILGL | 17169395 |
| p24 | RFY_299-314_ (RFYKTLRAEQASQEVK) | FRDYVDRFYKTLRAEQASQE  YVDRFYKTLRAEQASQEV | 27111229, 20195518; 23793098, 21047960 |
| p24 | NAN_325-345_ (NANPDCKTILKALGPGATLEE) | NANPDCKTILKALGPAA | 21047960 |
| p24 | ALG_336-359_ (ALGPGATLEEMMTACQGVGGPGHK) | TILKALGPAATLEEMMTA  LEEMMTACQGVGGPGHK | 24741089;  24465991 |
| p24-p2-p7 | GPG_355-375_ (GPGHKARVLAEAMSQVTNPAT) | GHKARVLAEAMSQVTNSA | 21047960 |
| p24-p2-p7 | NPA_372-388_ (NPATIMIQKGNFRNQRKT) | TNSATIMMQRGNFRNQRK | 23793098, 18167642 |
| Protease | GGF_107-117_ (GGFIKVRQYDQ) | KMIGGIGGFIKVRQYDQISI | 21352200 |
| Protease | GPT_134-148_ (GPTPVNIIGRNLLTQ) | LVGPTPINIIGRNLLTQIGC | 21352200 |
| RT | SQL_153-166_ (SQLPISPIETVPVK) | NFPISPIETVPVKLR | 22792193 |
| RT | EGK_199-212_ (EGKISKIGPENPYN) | TEMEKEGKISKIGPE | 7539750 |
| RT | SSM_317-327_ (SSMTKILEPFR) | SPAIFQSSMTKILEP | 11287569, 14632751 |
| RT | EPF_324-337_ (EPFRKQNPDIVIYQ) | FRKQNPDIVIYQYMDDLYV | 14512540 |
| RT | WTV_407-419_ (WTVNDIQKLVGKL) | KDSWTVNDIQKLVGK | 10932158, 7539750 |
| RT | EPF_499-511_ (EPFKNLKTGKYAR) | QEPFKNLKTGKYAKM | 23028895 |
| RT | EFV_570-579_ (EFVNTPPLVK) | WEFVNTPPLVKLWYQ | 11287569, 25786238 |
| RT | YQL_582-593_ (YQLEKEPIVGAE) | WYQLEKEPIVGAETFYVDGAANR | 15755584 |
| RT | AET_592-606_ (AETFYVDGAASRETK) | EPIVGAETFYVDGAANRET | 9886381 |
| INT | SGY_796-812_ (SGYIEAEVIPAETGQET) | VTSGYIEAEVIPAET | 22792193 |
| INT | SMN_868-881_ (SMNKELKKIIGQVR) | ELKKIIGQVRDQAEHLK | 19165342 |
| INT | MAV_893-900_ (MAVFIHNH) | KTAVQMAVFIHNFKR | 14632751 |
| INT | AGE_911-931_ (AGERIVDIIATDIQTKELQKQ) | SAGERIVDIIATDIQTK | 18167642 |
| INT | GEG_960-979_ (GEGAVVIQDNSDIKVVPRRK) | LWKGEGAVVIQDNSDIKV | 18167642 |
| gp120 | KEY_171-179_ (KEYALFYKL) | ALFYKLDVVPID | 17117012 |
| gp120 | LDV­_179-192_ (LDVVPIDNNNTSYR) | YALFYKLDVVPIDNDNTSY | 23793098 |
| gp120, BG505 SOSIP | SEL_481-499_ (SELYKYKVVKIEPLGVAPT) | SELYLYKVVKIEPLGVAP | 17117012 |
| BG505 SOSIP | ETF_466-576_ (ETFRPGGGDMR) | ETFRPGGGDMRNNWR | 18195071 |
| Nef | AAS_32-55_ (AASRDLEKHGAITSSNTAATNAAC) | LEKHGAITSSNTAATNA | 19457989 |
| Nef | FPV_68-80_ (FPVTPQVPLRPMT) | VGFPVTPQVPLRPMTYKAAVDL  SHFLKEKGGL | 17600593 |
| Nef | EKG_93-108_ (EKGGLEGLIHSQRRQD) | HFLKEKGGLEGLIYSQKR | 28771107 |
| Rev | ERQ_47-61_ (ERQRQIHSISERILG) | RRRRWRERQRQIHSIS | 8573390 |
| Tat | LSK_69-86_ (LSKQPTSQSRGDPTGPKE) | HQASLSKQPTSQPRGD | 7686223 |

*Studies of induced memory responses in PLWH and vaccine studies using HIV-1 antigens in LANL 2018 Database.
