# Supplementary material for: A cell-free antigen processing system informs HIV-1 epitope selection and vaccine design
Source: J Exp Med. 2023 Apr 14;220(7):e20221654. doi: 10.1084/jem.20221654 (PMC10114365; doi:10.1084/jem.20221654)
Supplement: Table S3 — shows glycopeptides obtained from cell-free processing identified by GPQuest. [file JEM_20221654_TableS3.docx]

**Table S3: Glycopeptides Obtained from Cell-Free Processing Identified by GP-Quest**

| **Protein** | **Glycopeptide Sequence** | **Predicted Glycan Type** | **Glycan Structure** | **Morpheus Score^1^** |
| --- | --- | --- | --- | --- |
| JR-FL gp120 | LDVVPIDNN**[NTS]**YR | N-linked | N2H8F0S0G0 | 10.3017027357 |
|  |  |  |  | 10.2725285262 |
|  |  |  |  | 10.2710827626 |
| JR-FL gp120 | VVPIDNN**[NTS]**YRLIS | N-linked | N2H8F0S0G0 | 9.2296398171 |
| JR-FL gp120 | SD**[NFT]**NNAKTIIVQ | N-linked | N2H8F0S0G0 | 11.4139604159 |
|  |  |  |  | 11.4063199157 |
|  |  |  |  | 18.373783814 |
|  |  |  |  | 13.3554094682 |
|  |  |  | N2H7F0S0G0  *N3H2F4S0G0 | 17.3794603783 |
| JR-FL gp120 | EQFE**[NKT]**IVF**[NHS]**SGGD^2^ | N-linked | N2H8F0S0G0 | 10.3878828518 |
| JR-FL gp120 | EQFE**[NKT]**IVF**[NHS]**SGGDPEIVM(Ox)^2^ | N-linked | N2H8F0S0G0 | 10.2785202328 |
| JR-FL gp120 | EQFE**[NKT]**IVF**[NHS]**SGGDPEIVM(Ox)H^2^ | N-linked | N6H3F0S0G0 | 10.2890859294 |
| JR-FL gp120 | SELYKYKVVKIEPLGVAP**(T)**KAKRR | O-linked | N1H1F0S2G0 | 15.1711694645 |
|  |  |  |  | 7.1726756136 |
|  |  |  | N1H1F0S0G0 | 11.1629914574 |
|  |  |  | N1H1F0S1G0 | 9.17048419972 |
| JR-FL gp120 | LYKYKVVKIEPLGVAP**(T)**KAKRR | O-linked | N1H1F0S2G0 | 10.1658616166 |
| JR-FL gp120 | KYKVVKIEPLGVAP**(T)**KAKRR | O-linked | N1H1F0S2G0 | 17.1967109812 |
|  |  |  |  | 10.1961173789 |
| JR-FL gp120 | KYKVVKIEPLGVAP**(T)**KAK | O-linked | N1H1F0S1G0 | 21.3157413884 |
|  |  |  | N1H1F0S2G0 | 16.2947052688 |
|  |  |  | N1H1F0S0G0 | 9.30401275396 |
| BG505 SOSIP | EEEVMIRSE**[NIT]**NNAKN | N-linked | N1H0F0S0G0 | 12.1688867995 |
| BG505 SOSIP | EEEVM(Ox)IRSE**[NIT]**NNAKN | N-linked | N2H5F0S0G0 | 10.2222338204 |
|  |  |  |  | 17.2097274113 |
| BG505 SOSIP | ATW**[NET]**LGKVVKQ | N-linked | N2H9F0S0G0 | 12.3560726868 |
|  |  |  |  | 11.4248065512 |
|  |  |  | N6H3F0S0G0 | 11.4006072492 |

^1^From GPQuest; see Anal. Chem. 2015, 87, 10, 5181–5188. Glycopeptides with a Morpheus Score of >6.16 were considered hits.

^2^Glycosylation can occur on either site

Legend:

(Ox) = Oxidation, N = N-acetylhexosamine (HexNAc), H = hexose, F = fucose, S = sialic acid,

G = N-Glycolylneuraminic acid (Neu5GC), * = alternate glycan

Gray shading indicates DR1 binder, green shading indicates DM-resistant DR1 binder
