# Supplementary material for: A cell-free antigen processing system informs HIV-1 epitope selection and vaccine design
Source: J Exp Med. 2023 Apr 14;220(7):e20221654. doi: 10.1084/jem.20221654 (PMC10114365; doi:10.1084/jem.20221654)
Supplement: Table S4 — shows select peptides identified from cell-free processing. [file JEM_20221654_TableS4.docx]

**Table S4: Select Peptides Identified from Cell-Free Processing**

| **Abbreviation**  **Peptide ID** | **Sequence** | **AA Position** | **NA Position** |
| --- | --- | --- | --- |
| Gag-SGG_9-20_ | SGGELDKWEKIR | Gag(9-20) | 814-849 |
| Gag-ASR_37-50_ | ASRELERFAVNPGLL* | Gag(37-51) | 898-942 |
| Gag-TSE_53-69_ | TSEGCRQILGQLQPSLQ | Gag(53-69) | 946-996 |
| Gag-TGS_70-81_ | TGSEELRSLYNT | Gag(70-81) | 997-1032 |
| Gag-QNY_130-151_ | QNYPIVQNIQGQMVHQAISPRT^$^ | Gag (130-151) | 1177-1242 |
| Gag-PIV_133-150_ | PIVQNLQGQMVHQAISPR | Gag(133-150) | 1186-1242 |
| Gag-SPE_165-176_ | SPEVIPMFSALS^$^ | Gag(165-176) | 1282-1317 |
| Gag-SAL_173-187_ | SALSEGATPQDLNTM | Gag(173-187) | 1306-1350 |
| Gag-EGA_177-199_ | EGATPQDLNTMLNTVGGHQAAMQ | Gag(177-199) | 1318-1386 |
| Gag-ETI_203-220_ | ETINEEAAEWDRLHPVHA | Gag(203-220) | 1396-1449 |
| Gag-EEA_207-227_ | EEAAEWDRLHPVHAGPIAPGQ | Gag(207-227) | 1408-1470 |
| Gag-AGP_220-244_ | AGPIAPGQMREPRGSDIAGTTSTLQ | Gag(220-244) | 1447-1521 |
| Gag-EPR_230-244_ | EPRGSDIAGTTSTLQ | Gag(230-244) | 1477-1521 |
| Gag-KRW_263-277_ | KRWIILGLNKIVRMY* | Gag(263-277) | 1576-1620 |
| Gag-FRD_293-312_ | FRDYVDRFYKTLRAEQASQE* | Gag(293-312) | 1666-1725 |
| Gag-RFY_299-314_ | RFYKTLRAEQASQEVK | Gag(299-314) | 1684-1731 |
| Gag-NAN_325-345_ | NANPDCKTILKALGPGATLEE | Gag(325-345) | 1762-1824 |
| Gag-ALG_336-359_ | ALGPAATLEEMMTACQGVGGPGHK | Gag(336-359) | 1795-1866 |
| Gag-GPG_355-375_ | GPGHKARVLAEAMSQVTNPAT | Gag(355-375) | 1852-1914 |
| Gag-EAM_365-380_ | EAMSQVTNPATIMIQK^$^ | Gag(365-380) | 1882-1929 |
| Gag-NPA_372-388_ | NPATIMIQKGNFRNQRT | Gag(372-388) | 1903-1953 |
| Pol-QRP_63-77_ | QRPLVTIKIGGQLKE* | Pol(63-77) | 2271-2315 |
| Pol-GPT_134-148_ | GPTPVNIIGRNLLTQ | Pol(134-148) | 2484-2528 |
| Pol-SQL_153-166_ | SQLPISPIETVPVK | Pol(153-166) | 2541-2582 |
| Pol-EGK_199-212_ | EGKISKIGPENPYN | Pol(199-212) | 2679-2720 |
| Pol-SVP_272-282_ | SVPLDEDFRKY*^ | Pol(272-282) | 2898-2930 |
| Pol-IPS_287-300_ | IPSINNETPGIRYQ^$^ | Pol(287-300) | 2943-2984 |
| Pol-ETP_293-307_ | ETPGIRYQYNVLPQG^$^ | Pol(293-307) | 2961-3005 |
| Pol-SSM_317-327_ | SSMTKILEPFR | Pol(317-327) | 3033-3065 |
| Pol-EPF_324-337_ | EPFRKQNPDIVIYQ | Pol(324-337) | 3054-3095 |
| Pol-VQP_396-404_ | VQPIVLPEK | Pol(396-404) | 3270-3296 |
| Pol-WTV_407-419_ | WTVNDIQKLVGKL^^#^ | Pol(407-419) | 3303-3341 |
| Pol-ASQ_422-436_ | ASQIYPGIKVRQLCK*^#^ | Pol(422-436) | 3348-3392 |
| Pol-EEA_452-466_ | EEAELELAENREILK | Pol(452-466) | 3438-3482 |
| Pol-LAE_458-478_ | LAENREILKEPVHGVYYDPSK | Pol(458-478) | 3456-3518 |
| Pol-EPF_499-511_ | EPFKNLKTGKYAR | Pol(499-511) | 3579-3617 |
| Pol-EFV_570-579_ | EFVNTPPLVK | Pol(570-579) | 3792-3821 |
| Pol-YQL_582-593_ | YQLEKEPIVGAE | Pol(582-593) | 3828-3863 |
| Pol-AET_592-606_ | AETFYVDGAASRETK | Pol (592-606) | 3858-3902 |
| Pol-LAW_688-705_ | LAWVPAHKGIGGNEQVDK^$^ | Pol (688-705) | 4146-4199 |
| Pol-HSN_731-745_ | HSNWRAMASDFNLPP* | Pol(731-745) | 4275-4319 |
| Pol-SGY_796-812_ | SGYIEAEVIPAETGQET | Pol(796-812) | 4470-4520 |
| Pol-AEV_801-818_ | AEVIPAETGQETAYFLLK | Pol(801-818) | 4485-4538 |
| Pol-AGI_848-867_ | AGIKQEFGIPYNPQSQGVIE | Pol(848-867) | 4626-4685 |
| Pol-IPY_856-875_ | IPYNPQSQGVIESMNKELKK^$^ | Pol(856-875) | 4650-4709 |
| Pol-SMN_868-881_ | SMNKELKKIIGQVR | Pol(868-881) | 4686-4727 |
| Pol-AGE_911-931_ | AGERIVDIIATDIQTKELQKQ | Pol(911-931) | 4815-4877 |
| Pol-TDI_921-937_ | TDIQTKELQKQITKIQN | Pol(921-937) | 4845-4895 |
| Pol-GEG_960-979_ | GEGAVVIQDNSDIKVVPRRK | Pol(960-979) | 4962-5021 |
| Vif-CEY_133-146_ | CEYQAGHNKVGSLQ | Vif(133-146) | 5437-5478 |
| Vif-SLQ_144-158_ | SLQYLALVALVAPKK* | Vif(144-158) | 5470-5514 |
| Tat-MEP_1-12_ | MEPVDPRLEPWK | Tat(1-12) | 5831-5866 |
| Tat-CYC_25-47_ | CYCKHCSYHCLVCFQTKGLGISY* | Tat(25-47) | 5903-5971 |
| Tat-LSK_69-86_ | LSKQPTSQSRGDPTGPKE | Tat(69-86) | 6035-6045, 8379-8421 |
| Env-EAT_47-67_ | EATTTLFCASDAKAYDTEVHN | Env(47-67) | 6363-6425 |
| Env-EEF_91-103_ | EEFNMWKNNMVEQ | Env(91-103) | 6495-6533 |
| Env-EHF_91-103_ | EHFNMWKNNMVEQ | Env(91-103) | 6495-6533 |
| Env-KVS_207-224_ | KVSFEPIPIHYCAPAGFA* | Env(207-224) | 6843-6896 |
| Env-YCA_217-227_ | YCAPAGFAILK | Env(217-227) | 6873-6905 |
| Env-EEE_267-283_ | EEEVMIRSENITNNAKN | Env(267-283) | 7023-7073 |
| Env-EEE_267-283_ | EEEVMIRSENITNNAKN+ | Env(267-283) | 7023-7073 |
| Env-SDN_274-287_ | SDNFTNNAKTIIVQ | Env(274-287) | 7044-7085 |
| Env-ETF_466-476_ | ETFRPGGGDMR | Env(466-476) | 7620-7652 |
| Env-SEL_481-499_ | SELYKYKVVKIEPLGVAPT | Env(481-499) | 7665-7721 |
| Env-KYK_485-499_ | KYKVVKIEPLGVAPT | Env(485-499) | 7677-7721 |
| Env-LGF_520-534_ | LGFLGAAGSTMGAAS^$^ | Env(520-534) | 7782-7826 |
| Env-LSG_545-557_ | LSGIVQQQSNLLR^$^ | Env(545-557) | 7857-7895 |
| Env-SGI_546-562_ | SGIVQQQSNLLRAPEAQ | Env(546-562) | 7860-7910 |
| Rev-ELL_11-27_ | ELLKTVRLIKFLYQSNP* | Rev(11-27) | 6000-6045, 8379-8383 |
| Rev-ERQ_47-61_ | ERQRQIHSISERILG | Rev(47-61) | 8441-8485 |
| Rev-ERI_57-74_ | ERILGTYLGRSAEPVPLQ | Rev(57-74) | 8471-8524 |
| Rev-SPQ_99-116_ | SPQILVESPTVLESGTKE | Rev(99-116) | 8597-8650 |
| Nef-EKG_93-108_ | EKGGLEGLIHSQRRQD | Nef(93-108) | 9073-9120 |
| Nef-LVP_145-163_ | LVPVEPDKIEEANKGENTS | Nef(145-163) | 9229-9285 |
| Nef-PEK_176-193_ | PEKEVLVWKFDSRLAFHH* | Nef(176-193) | 9322-9375 |

Legend:

*from literature; +glycosylated; ^added N’ terminal amino acid; #added C’ terminal amino acid; $discussed in text but not tested in patients. Peptide abbreviation (i.e. Nef-PEK_176-193_) consists of the protein from which the peptide derives (Nef), first three amino acids of the peptide (PEK), and the amino acid position relative to protein start in HXB2 (176-193). NA position refers to the nucleotide position encoding the peptide relative to genome start in HXB2.
