# Supplementary material for: A cell-free antigen processing system informs HIV-1 epitope selection and vaccine design
Source: J Exp Med. 2023 Apr 14;220(7):e20221654. doi: 10.1084/jem.20221654 (PMC10114365; doi:10.1084/jem.20221654)
Supplement: Table S5 — shows clinical characteristics of PLWH in study. [file JEM_20221654_TableS5.docx]

**Table S5: Clinical Characteristics of PLWH in Study**

| **ID** | **Gender** | **Age** | **cART** | **Viral load (copies/mL)** | **CD4 Count** | **CD8 Count** | **CD4 Nadir** | **Infection Time pre-cART (months)** | **Time on cART (yrs)** |
| --- | --- | --- | --- | --- | --- | --- | --- | --- | --- |
| 1351 | Male | 47 | BIC/FTC/TAF | <40 | 511 | 513 | 262 | 17 | 11 |
| 1716 | Male | 67 | FTC/TAF, RGV | <40 | 369 | 456 | 266 | 278 | 8 |
| 2056 | MTF transgender | 65 | FTC/TAF, TCV | <40 | 735 | 882 | 423 | 60 | 24 |
| 2238 | Male | 67 | BIC/FTC/TAF | <40 | 1063 | 736 | 538 | 1 | 13 |
| 2253 | Male | 68 | BIC/FTC/TAF | <40 | 424 | 387 | 359 | 71 | 13 |
| 2285 | Male | 48 | BIC/FTC/TAF | <40 | 940 | 816 | 395 | 1 | 13 |
| 2328 | Male | 50 | EGV/TAF/FTC/COBI | <40 | 712 | 828 | 420 | 1 | 12 |
| 2369 | Male | 67 | RPV/TAF/FTC | <40 | 837 | 295 | 490 | 89 | 11 |
| 3037 | Male | 60 | DOR, DRV, BIC/FTC/TAF | <40 | 581 | 1370 | 147 | 228 | 23 |
| 3641 | Male | 47 | EGV/TAF/FTC/COBI | <40 | 1402 | 555 | 666 | 21 | 5 |

*BIC: bictegravir; FTC: emtricitabine; TAF: tenofovir alafenamide; RGV: raltegravir; ABC: abacavir; 3TC: lamivudine; ETV: etravirine; TCV: dolutegravir ; EGV: elvitegravir; COBI: cobicistat; RPV: rilpivirine; DOR: doravirine; DRV: darunavir.
