# Supplementary material for: A cell-free antigen processing system informs HIV-1 epitope selection and vaccine design
Source: J Exp Med. 2023 Apr 14;220(7):e20221654. doi: 10.1084/jem.20221654 (PMC10114365; doi:10.1084/jem.20221654)
Supplement: Table S6 — shows sequences of proteins utilized in the cell-free processing system. [file JEM_20221654_TableS6.docx]

**Table S6: Sequences of Proteins Utilized in the Cell-Free Processing System**

| Protein | Strain | Sequence |
| --- | --- | --- |
| MyrMA | NL4.3 | **^M^**GARASVLSGGELDKWEKIRLRPGGKKQYKLKHIVWASRELERFAVNPGLLETSEGCRQILGQLQPSLQTGSEELRSLYNTIAVLYCVHQRIDVKDTKEALDKIEEEQNKSKKKAQQAAADTGNNSQVSQNY |
| p24 | HXB2 | PIVQNIQGQMVHQAISPRTLNAWVKVVEEKAFSPEVIPMFSALSEGATPQDLNTMLNTVGGHQAAMQMLKETINEEAAEWDRVHPVHAGPIAPGQMREPRGSDIAGTTSTLQEQIGWMTNNPPIPVGEIYKRWIILGLNKIVRMYSPTSILDIRQGPKEPFRDYVDRFYKTLRAEQASQEVKNWMTETLLVQNANPDCKTILKALGPAATLEEMMTACQGVGGPGHKARVLAHHHHHH |
| CA_SP1_NC | NL4.3 | MVSQNYPIVQNLQGQMVHQAISPRTLNAWVKVVEEKAFSPEVIPMFSALSEGATPQDLNTMLNTVGGHQAAMQMLKETINEEAAEWDRLHPVHAGPIAPGQMREPRGSDIAGTTSTLQEQIGWMTHNPPIPVGEIYKRWIILGLNKIVRMYSPTSILDIRQGPKEPFRDYVDRFYKTLRAEQASQEVKNWMTETLLVQNANPDCKTILKALGPGATLEEMMTACQGVGGPGHKARVLAEAMSQVTNPATIMIQKGNFRNQRKTVKCFNCGKEGHIAKNCRAPRKKGCWKCGKEGHQMKDCTERQAN |
| Protease | HXB2 | MFPQITLWQRPLVTIKIGGQLKEALLDTGADDTVLEEMSLPGRWKPKMIGGIGGFIKVRQYDQILIEICGHKAIGTVLVGPTPVNIIGRNLLTQIGCTLNFPISPIETVPVKLKPGMDGPKVKQWH |
| RT p66 (N-terminal 6X His tag) | HXB2 | MRGSHHHHHHGSQLPISPIETVPVKLKPGMDGPKVKQWPLTEEKIKALVEICTEMEKEGKISKIGPENPYNTPVFAIKKKDGTKWRKLVDFRELNKKTQDFWEVQLGIPHPAGLKKKKSVTVLDVGDAYFSVPLDEDFRKYTAFTIPSINNETPGIRYQYNVLPQGWKGSPAIFQSSMTKILEPFRKQNPDIVIYQYMDDLYVGSDLEIGQHRTKIEELRQHLLRWGLTTPDKKHQKEPPFLWMGYELHPDKWTVQPIVLPEKDSWTVNDIQKLVGKLNWASQIYPGIKVRQLCKLLRGTKALTEVIPLTEEAELELAENREILKEPVHGVYYDPSKDLIAEIQKQGQGQWTYQIYQEPFKNLKTGKYARMRGAHTNDVKQLTEAVQKITTESIVIWGKTPKFKLPIQKETWETWWTEYWQATWVPEWEFVNTPPLVKLWYQLEKEPIVGAETFYVDGAASRETKLGKAGYVTNRGRQKVVTLTDTTNQKTELQAIHLALQDSGLEVNIVTDSQYALGIIQAQPDQSESELVNQIIEQLIKKEKVYLAWVPAHKGIGGNEQVDKLVSAGIRKIL* |
| RT p51 (N-terminal 6X His tag) | HXB2 | MRGSHHHHHHGSQLPISPIETVPVKLKPGMDGPKVKQWPLTEEKIKALVEICTEMEKEGKISKIGPENPYNTPVFAIKKKDGTKWRKLVDFRELNKKTQDFWEVQLGIPHPAGLKKKKSVTVLDVGDAYFSVPLDEDFRKYTAFTIPSINNETPGIRYQYNVLPQGWKGSPAIFQSSMTKILEPFRKQNPDIVIYQYMDDLYVGSDLEIGQHRTKIEELRQHLLRWGLTTPDKKHQKEPPFLWMGYELHPDKWTVQPIVLPEKDSWTVNDIQKLVGKLNWASQIYPGIKVRQLCKLLRGTKALTEVIPLTEEAELELAENREILKEPVHGVYYDPSKDLIAEIQKQGQGQWTYQIYQEPFKNLKTGKYARMRGAHTNDVKQLTEAVQKITTESIVIWGKTPKFKLPIQKETWETWWTEYWQATWVPEWEFVNTPPLVKLWYQLEKEPIVGAETF* |
| Integrase (F185H/C280S) | NL4.3 | FLDGIDKAQEEHEKYHSNWRAMASDFNLPPVVAKEIVASCDKCQLKGEAMHGQVDCSPGIWQLDCTHLEGKVILVAVHVASGYIEAEVIPAETGQETAYFLLKLAGRWPVKTVHTDNGSNFTSTTVKAACWWAGIKQEFGIPYNPQSQGVIESMNKELKKIIGQVRDQAEHLKTAVQMAVFIHNHKRKGGIGGYSAGERIVDIIATDIQTKELQKQITKIQNFRVYYRDSRDPVWKGPAKLLWKGEGAVVIQDNSDIKVVPRRKAKIIRDYGKQMAGDDCVASRQDED |
| gp120 | JR-FL | MPMGSLQPLATLYLLGMLVASVLAVEKLWVTVYYGVPVWKEATTTLFCASDAKAYDTEVHNVWATHACVPTDPNPQEVVLENVTEHFNMWKNNMVEQMQEDIISLWDQSLKPCVKLTPLCVTLNCKDVNATNTTNDSEGTMERGEIKNCSFNITTSIRDEVQKEYALFYKLDVVPIDNNNTSYRLISCDTSVITQACPKISFEPIPIHYCAPAGFAILKCNDKTFNGKGPCKNVSTVQCTHGIRPVVSTQLLLNGSLAEEEVVIRSDNFTNNAKTIIVQLKESVEINCTRPNNNTRKSIHIGPGRAFYTTGEIIGDIRQAHCNISRAKWNDTLKQIVIKLREQFENKTIVFNHSSGGDPEIVMHSFNCGGEFFYCNSTQLFNSTWNNNTEGSNNTEGNTITLPCRIKQIINMWQEVGKAMYAPPIRGQIRCSSNITGLLLTRDGGINENGTEIFRPGGGDMRDNWRSELYKYKVVKIEPLGVAPTKAKRRVVQREKRGSLEVLFQGPGHHHHHH |
| SOSIP (gp140 ectodomain) | BG505 | MPMGSLQPLATLYLLGMLVASVLAAENLWVTVYYGVPVWKDAETTLFCASDAKAYETEKHNVWATHACVPTDPNPQEIHLENVTEEFNMWKNNMVEQMHTDIISLWDQSLKPCVKLTPLCVTLQCTNVTNNITDDMRGELKNCSFNMTTELRDKKQKVYSLFYRLDVVQINENQGNRSNNSNKEYRLINCNTSAITQACPKVSFEPIPIHYCAPAGFAILKCKDKKFNGTGPCPSVSTVQCTHGIKPVVSTQLLLNGSLAEEEVMIRSENITNNAKNILVQFNTPVQINCTRPNNNTRKSIRIGPGQAFYATGDIIGDIRQAHCNVSKATWNETLGKVVKQLRKHFGNNTIIRFANSSGGDLEVTTHSFNCGGEFFYCNTSGLFNSTWISNTSVQGSNSTGSNDSITLPCRIKQIINMWQRIGQAMYAPPIQGVIRCVSNITGLILTRDGGSTNSTTETFRPGGGDMRDNWRSELYKYKVVKIEPLGVAPTRCKRRVVGRRRRRRAVGIGAVFLGFLGAAGSTMGAASMTLTVQARNLLSGIVQQQSNLLRAPEAQQHLLKLTVWGIKQLQARVLAVERYLRDQQLLGIWGCSGKLICCTNVPWNSSWSNRNLSEIWDNMTWLQWDKEISNYTQIIYGLLEESQNQQEKNEQDLLALD |
| Vif | HXB2 | MENRWQVMIVWQVDRMRIRTWKSLVKHHMYVSGKARGWFYRHHYESPHPRISSEVHIPLGDARLVITTYWGLHTGERDWHLGQGVSIEWRKKRYSTQVDPELADQLIHLYYFDCFSDSAIRKALLGHIVSPRCEYQAGHNKVGSLQYLALAALITPKKIKPPLPSVTKLTEDRWNKPQKTKGHRGSHTMNGH |
| Nef | HXB2 | MGGKWSKSSVIGWPTVRERMRRAEPAADRVGAASRDLEKHGAITSSNTAATNAACAWLEAQEEEEVGFPVTPQVPLRPMTYKAAVDLSHFLKEKGGLEGLIHSQRRQDILDLWIYHTQGYFPDQNYTPGPGVRYPLTFGWCYKLVPVEPDKIEEANKGENTSLLHPVSLHGMDDPEREVLEWRFDSRLAFHHVARELHPEYFKN |
| Rev | HXB2 | GAMAGRSGDSDEELIRTVRLIKLLYQSNPPPNPEGTRQARRNRRRRWRERQRQIHSISER**I**L**G**TYLGRSAEPVPLQLPPLERLTLDCNEDCGTSGTQGVGSPQILVESPTVLESGTKE* |
| Tat | IIIB | MEPVDPRLEPWKHPGSQPKTACTNCYCKKCCFHCQVCFMTKALGISYGRKKRRQRRRAHQNSQTHQASLSKQPTSQSRGDPTGPKE*KKKVERETETDPFD* |

**^M^** =Myristoyl group
